# Using gas mixtures of CO, CO_2_ and H_2_ as microbial substrates: the do's and don'ts of successful technology transfer from laboratory to production scale

**DOI:** 10.1111/1751-7915.13270

**Published:** 2018-05-14

**Authors:** Ralf Takors, Michael Kopf, Joerg Mampel, Wilfried Bluemke, Bastian Blombach, Bernhard Eikmanns, Frank R. Bengelsdorf, Dirk Weuster‐Botz, Peter Dürre

**Affiliations:** ^1^ Institute of Biochemical Engineering University of Stuttgart Allmandring 31 70569 Stuttgart Germany; ^2^ BASF SE Bio‐Process Development Carl‐Bosch‐Str. 38 67056 Ludwigshafen Germany; ^3^ BRAIN AG Darmstädter Straße 34‐36 64673 Zwingenberg Germany; ^4^ Evonik Technology and Infrastructure GmbH Process Technology & Engineering Rodenbacher Chaussee 4 63457 Hanau‐Wolfgang Germany; ^5^ Institute of Microbiology and Biotechnology University of Ulm Albert‐Einstein‐Allee 11 89081 Ulm Germany; ^6^ Department of Mechanical Engineering Institute of Biochemical Engineering Technical University of Munich Boltzmannstr. 15 85748 Garching Germany

## Abstract

The reduction of CO_2_ emissions is a global effort which is not only supported by the society and politicians but also by the industry. Chemical producers worldwide follow the strategic goal to reduce CO_2_ emissions by replacing existing fossil‐based production routes with sustainable alternatives. The smart use of CO and CO_2_/H_2_ mixtures even allows to produce important chemical building blocks consuming the said gases as substrates in carboxydotrophic fermentations with acetogenic bacteria. However, existing industrial infrastructure and market demands impose constraints on microbes, bioprocesses and products that require careful consideration to ensure technical and economic success. The mini review provides scientific and industrial facets finally to enable the successful implementation of gas fermentation technologies in the industrial scale.

## Introduction

Since at least December 2015, when an overwhelming majority of nations worldwide agreed on signing the Paris Climate Agreement, scientific theories of climate change attributing the increasing man‐made release of CO_2_ a dominating role started to drive political and economic decision‐making (Philip, 2018). Further supported by the inherent fear of diminishing fossil resources, oil‐based chemical industries worldwide began to develop future scenarios for ensuring the current product portfolios, aiming for zero‐CO_2_ emission strategies. At best, future processes should not only prevent non‐necessary CO_2_ emissions, preferred production technologies should even incorporate CO_2_ (Bengelsdorf and Dürre, 2017), thereby contributing to the climate goals and preventing costly payments for CO_2_ certificates.

One strategy to prevent CO_2_ emissions is the implementation of the so‐called circular economy, that is the use of sugar contents in lignocellulosic feedstocks such as agri‐residues, agri‐processing by‐products and energy crops for the microbial production of value‐added products such as biofuels or fine chemicals (Liguori and Faraco, 2016). Expectations formulated in the US Energy Independence and Security Act (2007) specified 35 billion gallons of ethanol equivalents to be used in 2022 as a strategic goal. However, the technically and economically successful implementation of such processes has revealed to be very challenging, still requiring progress. Handling and use of the lignin fraction including energy management is one of the challenges.

Biomass gasification either conventional (Griffin and Schultz, 2012) or via fast pyrolysis (Pfitzer *et al*., 2016; Arnold *et al*., 2017) is very well suited to use lignin‐containing sources such as wood or even municipal wastes for the production of CO and H_2_ containing gases, further called ‘syngas’ for simplification (LanzaTech, 2017). Such compositions vary between 30–60% CO, 25–30% H_2_, 0–5% CH_4_, 5–1 5% CO_2_, may contain other impurities such as H_2_S, NH_3_ and depend heavily on the source. A key transformation is the so‐called water/gas‐shift (WGS) reaction that converts CO and H_2_O into CO_2_ and H_2_ under high pressure and high temperatures (> 600°C) thus representing a source of H_2_ production. Besides for heat and power supply, syngas is applied in Fischer–Tropsch (FT) synthesis for the production of naphtha‐like mixtures, diesel, methanol or even ethanol. However, such processes are not only energy‐intensive (20–300 bar, 200–350°C), and they also require the removal of impurities like tars to protect the Rh catalysts. Furthermore, optimum FT conversions are only achieved if the preferred ratio H_2_ to CO of > 2 is installed (Abubackar *et al*., 2011; Griffin and Schultz, 2012).

Interesting enough, nature provides a whole bunch of fermentative microbes that may grow on H_2_, CO and CO_2_ compositions via hydrogenesis, methanogenesis or acetogenesis (Latif *et al*., 2014; Diender *et al*., 2015). The latter are particularly promising for industrial application as they make use of the reductive acetyl‐CoA pathway (Dürre, 2016). In a nutshell, CO and H_2_ serve as electron donors enabling the growth on CO_2_ and H_2_, or CO, or CO and H_2_ to produce mixtures of acetate, ethanol, 2,3 butanediol, etc. (Daniell *et al*., 2012). Accordingly, not only syngas, but also other off‐gas compositions, for example from coke oven plants and steel industry, may provide valuable electron sources for the microbes. Thereby, the CO dehydrogenase (CODH) is the enabling enzyme and the biological equivalent to the harsh technical approach, however, working under moderate, anaerobic conditions. Furthermore, feasible H_2_/CO ratios are much more flexible than in Fischer–Tropsch (FT) processes (Munasinghe and Khanal, 2010), albeit cellular performance might suffer from gas impurities such as sulfur dioxide or hydrogen sulfide. Nevertheless, such processes offer the potential to be economically superior to conventional FT approaches (Griffin and Schultz, 2012). Nevertheless, some inherent drawbacks still hamper the success of acetogenic production processes. Engineering challenges are mirrored by the poor water solubilities and low Henry constants of CO and H_2_ which are about 30 and 1.6 mg l^−1^ (of pure gases), and 27.1 and 1.6 mg bar^−1^ respectively.

Aside from the anaerobic, acetogenic bacteria, the aerobic carboxydotrophic bacteria are promising candidates for microbial production of value‐added products from gases. These organisms are able to grow chemolithoautotrophically on CO or syngas by use of the reductive pentose phosphate pathway for anabolism and O_2_ as final electron acceptor in energy metabolism. Aerobic CO oxidation is more exotherm and allows higher ATP generation than anaerobic fermentation with CO, and therefore, the production of complex and more ATP‐intensive products should be feasible. However, molecular toolboxes for carboxydotrophic bacteria are missing, and thus, strain engineering is also still challenging.

This review not only provides biological and technical fundamentals for using acetogenic or aerobic, carboxydotrophic bacteria in gas fermentation, it also outlines the industrial point of view integrating such bioprocesses into both existing infrastructure and existing value‐added chains (VACs). Thereof, conclusions will be drawn to make zero‐CO_2_ initiatives an environmental and economic success.

## The *status quo* in chemical industry

The production landscape in chemical industry is often organized in value‐added chains (VACs), each leading from basic raw materials to molecules of interest via specific, often multifunctional intermediates. Currently, most VACs are based on fossil raw materials deploying homogeneous and heterogeneous chemical catalysis as major synthesis technologies. However, driven by competitiveness and the mindset of zero‐CO_2_ emission processes, the use of alternative raw materials, such as renewable raw materials (RRM), is gaining importance.

Nevertheless, such processes necessarily need to offer competitive products, produced via efficient approaches delivering material with at least the same quality and performance as produced from fossil resources. Irrespective of the raw material used and the chemical or biochemical conversions applied, the following constraints need to be fulfilled for each industrial process:

- New processes to produce existing products (drop‐in) need to show the potential to reach the efficiency and economic performance of mature traditional production routes;
- Product and process specifications need to consider the variability in raw material and the consequences of biosynthesis;
- Methods, techniques and equipment to economically handle large amounts of aqueous systems and purify molecules of interest in an aqueous environment are indispensable;
- In the long run, and taking into account both, economic reality and depreciation of existing assets, current VACs might need to be altered to take full advantage of bio‐based processes which might lead to new molecules.

On the other hand, gaseous substrates such as H_2_, CO and CO_2_ offer general benefits such as global availability, large capacities, no interference with food or feed (based on gas generation from fossil resources or wastes), predictable pricing through well‐known mechanisms, full metabolic usability, potentially less impurities derived from non‐reactive carbon, good storability, reduced risk of infection, access from waste streams and enabler for circular economy considerations.

## Products of interest

Driven by the inherent potential of gas fermentation, chemical industry should have a key interest to evaluate gaseous substrates for production purposes for intermediates as well as for performance molecules. Examples of the first are compounds such as 3‐hydroxypropionic acid, succinic acid, itaconic acid, 1,4‐butanediol, isobutene, 1‐octanol, methyl methacrylate (MMA), butadiene, fatty acids, amino acids. Examples of the second are highly functionalized short‐ and medium‐chain molecules, active pharma ingredients, vitamins, industrial enzymes, proteins, etc. It is particularly the first group that feels the strongest market pressure of success which is mirrored by the competition with traditional, fossil‐based production technologies, the high cost pressure and strict specifications given by chemical VACs and applications. On the other side, physicochemical properties of the compounds are often well known which offers a broad range of feasible downstream processing (DSP) unit operations, including non‐aqueous media in medium‐ to large‐size production volumes.

**Figure 1:**
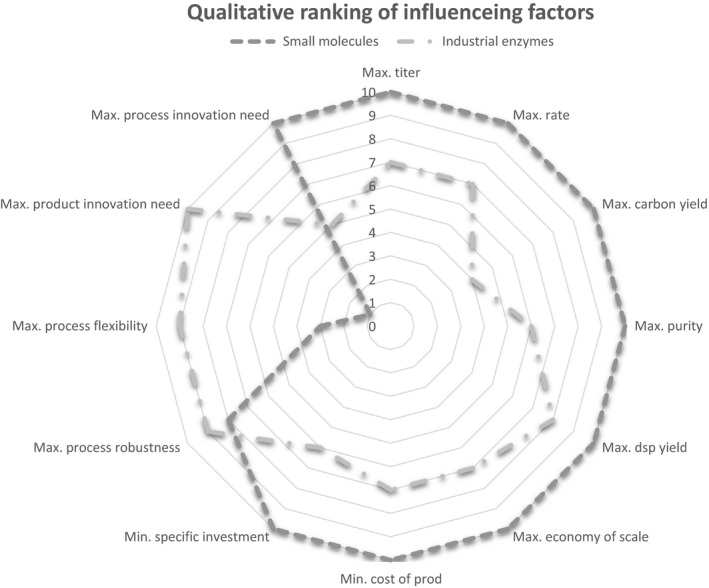
Crucial properties of success are given for intermediates of VACs (indicated as small molecules) and performance molecules (indicated as industrial enzymes). The ranking from 0 to 10 represents a qualitative measure.

To be successful, intermediates and performance molecules need to fulfil different criteria which mirror the individual production and market scenarios. Given the typically large market volumes of intermediates, also called commodities, of > 100.000 tons per year, maximum product concentrations (>100 g kg^−1^), production rates (> 4 g kg^−1^ h^−1^), conversion and downstream processing yields, and highest product purity are crucial properties. On the other hand, high‐process flexibility and product innovation levels are characteristic for performance molecules (see Fig. 1). As a consequence, gaseous substrates are particularly attractive for the production of commodities and are in the focus of current studies.

## Economic constraints of attractive gaseous substrates

The economic success of a gas fermentation‐based process will depend on several factors. First and foremost, the nature of the gaseous substrate is key. Gas mixtures of CO, CO_2_ and H_2_ are favourable due the inherent energy content of CO and H_2_. Off‐gases containing exclusively CO_2_ (> 95%) are less attractive, however, available in large amounts from power plants or large‐scale fermentations (e.g. bioethanol production). Unlike syngas, CO_2_‐rich waste streams are inert and CO_2_ activation requires reductive energy. Therefore, economic viability is crucially dependent on the availability of cheap and renewable electricity which has to be considered as the other feedstock besides CO_2_.

Hydrogen is a versatile energy transport molecule that can be stored using existing technologies (compression, hydrogen–gas grids). Currently, hydrogen formation by the alkaline technology or by the more preferable Proton‐Exchange‐Membrane (PEM) technology is still not cost competitive to the alternative Steam‐Methane‐Reformation (SMR) process that uses natural gas (methane). The latter liberates CO_2_, still, in some cases, the stoichiometry of the overall production process allows for a net fixation of CO_2_. In these cases, SMR can serve as a bridging technology towards a fully renewable process that is based on the flexible PEM technology. PEM hydrolyzers are costly, but prices are expected to fall by more than 30% within the next years (Bertuccioli *et al*., 2014 ). Considering that electricity prices dominate hydrogen–feedstock costs, product formation from CO_2_ will be most attractive during periods of surplus wind‐ and solar energy. Coupling of the energy providing industries with chemical industries (sector coupling) will be of increasing importance. However, existing regulatory frameworks have to be considered which might hamper cross‐industry synergies.

Molecular oxygen is the other product of the water splitting reaction and will be available in high purities and in large amounts, thus credits from O_2_ production can lower the hydrogen production costs. The often asynchronous availability of the three gases CO_2_, H_2_ and O_2_ asks for smart gas storage systems, but compressing gases is an established, however, energy‐demanding technique. Again, availability of cheap energy enters centre stage.

The common notion about CO_2_ is a valueless waste stream instead of a valuable feedstock. The revenues from the European Emissions Trading System (ETS certificates, about 7 €/ton) currently underpin this notion. They are too low to have a positive impact on business models that aim for CO_2_ upgrading (Pérez‐Fortes *et al*., 2016). However, depending on its purity, CO_2_ already has some markets mainly in food industries (beverages, green houses), thereby generating revenues with comparably little need of costly upfront investments (CAPEX, e.g., for gas stripping and compression technologies). CO_2_ finds further applications as co‐polymer in chemical synthesis, as shielding gas, extinguishing agent and as coolant. Existing CO_2_ markets compete for the feedstock with the novel VACs to be established. However, CO_2_ availability at point sources (steel mills, power stations, large‐scale biological fermentations) outnumbers the current demand by orders of magnitude (Mikkelsen *et al*., 2010). Accordingly, low CO_2_ feedstock prices will stay rather stable in the foreseeable future. For economic reasons, point sources of CO_2_ should be located at sites where renewable energy is available in sufficient amounts, for example Iceland (geothermal energy), Morocco (solar) or Norway (wind). Liquification of CO_2_ is an established technology, and long‐distance transport is economically viable for other gases (e.g. LNG, liquified natural gas). Unlike syngas, CO_2_ is available from the atmosphere in almost unlimited quantities. Average annual anthropogenic CO_2_ emissions are in the range of 35 ‐ 40 Gt, and CO_2_ emissions from fossil fuels and industry are expected to grow (Le Quéré *et al*., 2017). Consequently, direct air‐capture of CO_2_ in combination with gas fermentation at preferred sites becomes an interesting future option to enable economically viable production processes at currently unrecognized places. First pilot and commercial processes for direct air‐capture have been implemented but lack economic competitiveness at present (Climeworks, Skytree). With regard to syngas, gas fermentation facilities will have to be located next to the syngas‐emitting point source, and the first pilot‐ and demonstration‐scale processes are implemented (Lanzatech, 2017).

## The industrial wish list of microbial properties

Any microbial strain used in industrial applications needs to meet basic expectations. Properties such as high product tolerance, robustness with respect to harsh production conditions, genetic stability and high substrate uptake and conversion rates are highly appreciated aside from the expected high conversion yields. Regarding gas fermentations, the wish list can even be extended: the gaseous substrate might contain oxygen along with other trace toxins (e.g. sulfide) finally accumulating in the nutrient broth. Accordingly, oxygen tolerance is desirable, although somewhat contradicting with the native endowments of anaerobic acetogenic bacteria that are often applied. Similarly, microbes should tolerate elevated substrate levels (CO_2_, H_2_, CO), which are often mandatory in technical processes. Also, pH drops should be accepted because acid production typically coincides with the production of the product of choice.

## Fermentative metabolism of CO, H_2_ and CO_2_ containing gases

**Figure 2:**
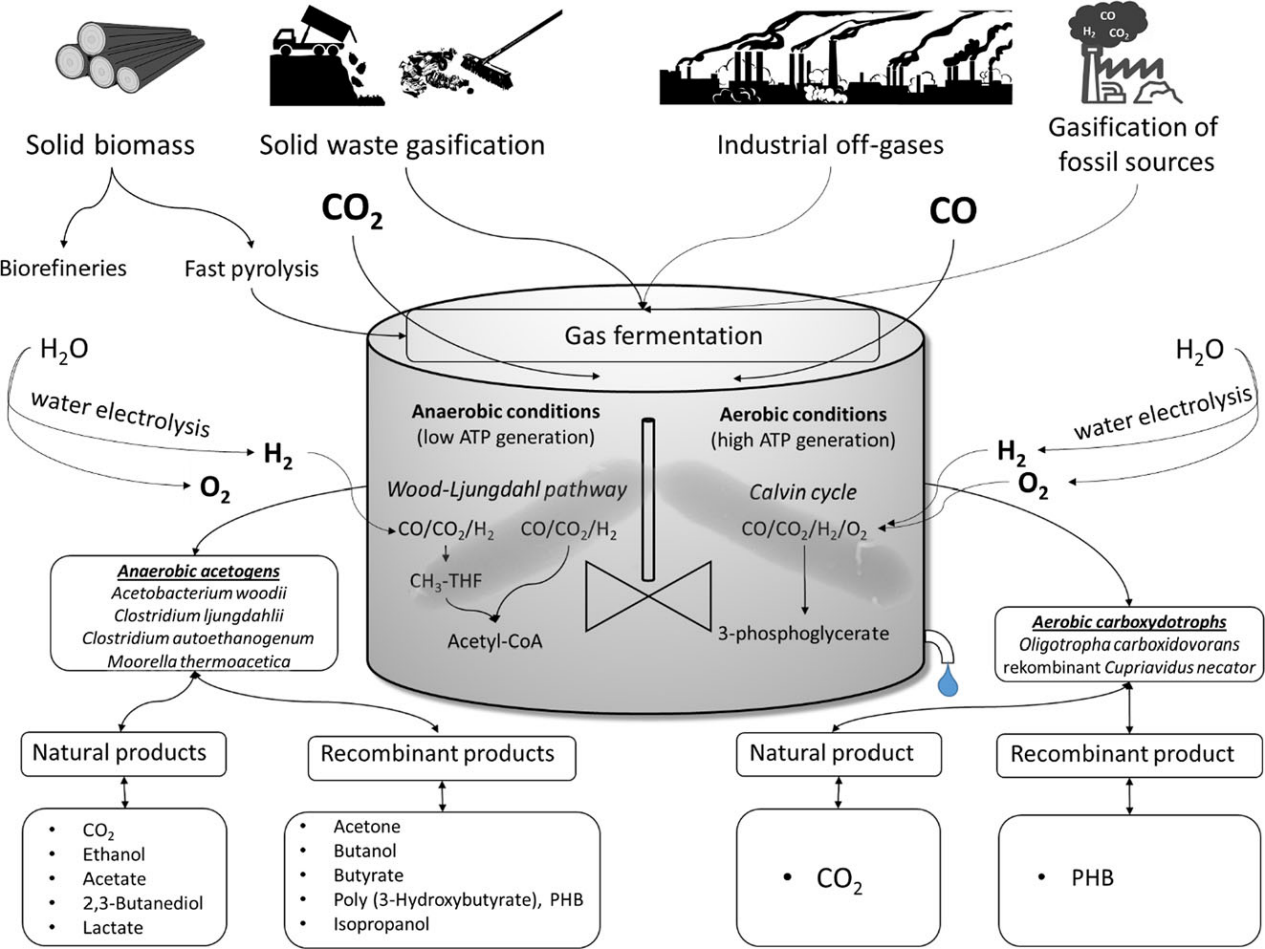
Syngas sources, anaerobic and aerobic syngas fermentation, model organisms involved, and natural and recombinant products.

Anaerobic acetogenic bacteria use the so‐called Wood–Ljungdahl pathway for fixation of CO_2_ or CO (Bengelsdorf *et al*., 2018). This pathway consists of two parts: the methyl and the carbonyl branch. In the former, a molecule of CO_2_ is reduced to formate (in case of CO as a carbon source, this is first oxidized to CO_2_) (Fig. 2). Formate is coupled to the coenzyme tetrahydrofolate, thereby hydrolysing one ATP into ADP and inorganic phosphate. Then, the C_1_ unit is successively reduced to methyl‐tetrahydrofolate, and the methyl group is transferred to an iron–sulfur–corrinoid protein. In the carbonyl branch, a molecule of CO_2_ is reduced to CO, using reduced ferredoxin, by the enzyme acetyl‐CoA synthase/CO dehydrogenase, which then also combines the methyl group of the iron–sulfur–corrinoid protein, a CoA moiety and the carbonyl group into acetyl‐CoA. This intermediate is further metabolized into acetate, yielding one ATP in the acetate kinase reaction. Thus, no substrate level‐phosphorylated ATP is left for growth and biosynthetic reactions. Acetogens obtain additional energy from ion gradients, either generated by the so‐called Rnf complex (a reduced ferredoxin: NAD^+^ oxidoreductase, producing thereby either a proton or a Na^+^ gradient) or the Ech complex, which oxidizes reduced ferredoxin, reduces protons, thereby producing hydrogen and translocates protons across the cytoplasmic membrane (Schuchmann and Müller, 2014). Acetogens that produce ethanol in addition to acetate do so using an aldehyde: ferredoxin oxidoreductase, which converts acetate and reduced ferredoxin to acetaldehyde that, in a further enzymatic reaction, is reduced to ethanol. This way, the bacteria are still able to generate ATP from acetate formation. Reducing equivalents are usually produced by bifurcating hydrogenases that oxidize two H_2_ and reduce both, ferredoxin and NAD^+^.

Numerous acetogens are currently known (Table 1). However, only few serve as model organisms and industrial workhorses, that is *Acetobacterium woodii*,* Clostridium ljungdahlii*,* Moorella thermoacetica* and *Clostridium autoethanogenum*. Although the current scientific and industrial interest mostly focuses on the autotrophic features, it must be mentioned that all acetogens known are also able to use a number of heterotrophic substrates. *A. woodii* was isolated in 1977, when the very first described autotrophic acetogen, that is *C. aceticum* was still considered to be lost (Balch *et al*., 1977). *C. aceticum* was only rediscovered in 1980 and later completely sequenced (Braun *et al*., 1981; Poehlein *et al*., 2015a,b,c). *A. woodii* does not use CO as a substrate, but is very efficient in fermenting CO_2_/H_2_ gas mixtures. It is now considered as the model acetogen for sodium bioenergetics. Energy conservation is based on a Rnf complex that exports Na^+^ ions and a sodium‐dependent ATPase that imports Na^+^ ions for ATP generation (Müller *et al*., 2001; Biegel and Müller, 2010; Hess *et al*., 2013). The organism is genetically accessible since more than 20 years (Strätz *et al*., 1994), the complete genome sequence is known (Poehlein *et al*., 2012), *A. woodii* can be grown in bioreactors with continuous gas supply, and recombinant strains for non‐natural product formation (acetone) have been constructed and tested (Straub *et al*., 2014; Hoffmeister *et al*., 2016; Kantzow and Weuster‐Botz, 2016).

**Table 1:** Acetogens and their major characteristics

Organism	Substrate	Products/Methanol utilization (yes or no)	Optimal growth temperature [°C]	Optimal pH	Doubling time (autotrophic) [h]	Genome accession number	References
*Acetitomaculum ruminis* DSM 5522	H_2_ + CO_2_, CO	Acetate/no	37–42	6.8	H_2_ + CO_2_: 2.1	FOJY00000000	(Greening and Leedle, 1989)
*Acetoanaerobium noterae* ATCC 35199	H_2_ + CO_2_	Acetate/no	37	7.6–7.8	H_2_ + CO_2_: 27	FUYN00000000	(Sleat *et al*., 1985)
*Acetobacterium bakii* DSM 8239	H_2_ + CO_2_, CO	Acetate/yes	20	6.5		LGYO01000000	(Kotsyurbenko *et al*., 1995 Hwang *et al*., 2015)
*Acetobacterium carbinolicum* DSM 2925	H_2_ + CO_2_	Acetate/yes	27	7.0–7.2			(Eichler and Schink, 1984; Schuppert and Schink, 1990)
*Acetobacterium dehalogenans* DSM 11527	H_2_ + CO_2_, CO	Acetate/yes	25	7.3–7.7		AXAC01000000	(Traunecker *et al*., 1991)
*Acetobacterium fimetarium* DSM 8238	H_2_ + CO_2_, CO	Acetate/no	30	7.5			(Kotsyurbenko *et al*., 1995)
*Acetobacterium malicum* DSM 4132	H_2_ + CO_2_	Acetate/no	30	7.5–8.0			(Tanaka and Pfennig, 1988)
*Acetobacterium paludosum* DSM 8237	H_2_ + CO_2_, CO	Acetate/yes	20	7.0			Kotsyurbenko *et al*., 1995)
*Acetobacterium tundrae* DSM 9173	H_2_ + CO_2_, CO	Acetate/yes	20	7.0			(Simankova *et al*., 2000)
*Acetobacterium wieringae* DSM 1911	H_2_ + CO_2_	Acetate/no	30	7.6		LKEU00000000	(Braun and Gottschalk, 1982 Poehlein *et al*., 2016)
*Acetobacterium woodii* DSM 1030	H_2_ + CO_2_	Acetate/yes	30	7.6		CP002987	(Balch *et al*., 1977; Bache and Pfennig, 1981; Genthner and Bryant, 1982; Schink and Stieb, 1983; Poehlein *et al*., 2012)
*Acetohalobium arabaticum* DSM 5501	H_2_ + CO_2_, CO	Acetate/no	38–40	7.6–8.0		CP002105	(Zhilina and Zavarzin, 1990 Sikorski *et al*., 2010)
*Acetonema longum* DSM 6540	H_2_ + CO_2_	Acetate, butyrate/n.r.	30–33	7.8		AFGF01000000	(Kane and Breznak, 1991 Chen *et al*., 2011)
*Alkalibaculum bacchi* DSM 22112	H_2_ + CO_2_, CO	Acetate, CO_2_, ethanol/yes	37	8.0–8.5			(Allen *et al*., 2010)
*Blautia coccoides* GA‐1^a^ n.d.	H_2_ + CO_2_	Acetate/n.r.	37^b^	7.0^c^			(Kaneuchi *et al*., 1976; Liu *et al*., 2008, 2015)
*Blautia hydrogenotrophica* DSM 10507	H_2_ + CO_2_	Acetate/n.r.	35–37	6.6		CYXL01000000	(Bernalier *et al*., 1996; Liu *et al*., 2008)
*Blautia producta* U‐1^a^ DSM 3507	H_2_ + CO_2_, CO	Acetate/no	37		CO: 1.5‐3	AUUC00000000	(Lorowitz and Bryant, 1984; Geerligs *et al*., 1987; Liu *et al*., 2008)
*Blautia schinkii* DSM 10518	H_2_ + CO_2_	Acetate/no	39	6.5–7.0		JNKJ01000000	(Rieu‐Lesme *et al*., 1996; Liu *et al*., 2008)
*‘Butyribacterium methylotrophicum’* c DSM 3468	H_2_ + CO_2_, CO (after adaption)	Acetate, ethanol, butyrate, butanol/yes	37–40	7.5	CO: 13.9	MIMZ00000000	(Zeikus *et al*., 1980; Lynd *et al*., 1982 Bengelsdorf *et al*., 2016b)
*Clostridium aceticum* DSM 1496	H_2_ + CO_2_, CO	Acetate/no	30	8.3	H_2_ + CO_2_: 20‐25	CP009687‐CP009688	(Wieringa, 1936; Lux and Drake, 1992; Braun *et al*., 1981 Poehlein *et al*., 2015c)
*‘Clostridium autoethanogenum’* c DSM 10061	H_2_ + CO_2_, CO	2,3‐butanediol, acetate, ethanol/no	37	5.8–6.0	CO: 4	CP006763	(Abrini *et al*., 1994; Köpke *et al*., 2011 Brown *et al*., 2014)
*Clostridium carboxidivorans* DSM 15243	H_2_ + CO_2_, CO	Acetate, ethanol, butyrate, butanol/no	38	5.0–7.0	CO: 6.3, H_2_ + CO_2_: 8.3	CP011803‐CP011804	(Liou *et al*., 2005)
*‘Clostridium coskatii’* c ATCC PTA‐10522	H_2_ + CO_2_, CO	Acetate, ethanol/no	37	5.8–6.5		LROR00000000	(Zahn and Saxena, 2012 Bengelsdorf *et al*., 2016a;
*Clostridioides difficile* 630^a^ DSM 27543	H_2_ + CO_2_	Acetate/n.r.	37^b^	5.9^b^		CP010905	Köpke *et al*., 2013 Riedel *et al*., 2015)
*Clostridium drakei* DSM 12750	H_2_ + CO_2_, CO	Acetate, ethanol, butyrate/no	30–37	5.4–7.5	CO: 8.3, H_2_ + CO_2_: 5.0	JIBU02000000	(Küsel *et al*., 2000; Liou *et al*., 2005; Gößner *et al*., 2008; Jeong *et al*., 2014)
*Clostridium formicaceticum* DSM 92	CO	Acetate, formate/yes	37	8.1	CO: 10	CP020559	(Andreesen *et al*., 1970; Lux and Drake, 1992 Karl *et al*., 2017)
*Clostridium ljungdahlii* DSM 13528	H_2_ + CO_2_, CO	2,3‐butanediol, acetate, ethanol/no	37	6.0	CO: 3.8	CP001666	(Tanner *et al*., 1993; Köpke *et al*., 2010, 2011)
*Clostridium magnum* DSM 2767	H_2_ + CO_2_	Acetate/yes	30–32	7.2		LWAE00000000	(Schink, 1984; Schink, 1991 *;* Uhlig *et al*., 2016)
*Clostridium methoxybenzovorans* DSM 12182	H_2_ + CO_2_	Acetate, formate/yes	37	7.4		ATXD01000000	(Mechichi *et al*., 1999)
*‘Clostridium ragsdalei’* c DSM 15248	H_2_ + CO_2_, CO	2,3‐butanediol, acetate, ethanol/n.r.	37		CO: 4	LROS00000000	(Huhnke *et al*., 2008; Köpke *et al*., 2011 Bengelsdorf *et al*., 2016a)
*Clostridium scatologenes* DSM 757	H_2_ + CO_2_, CO	Acetate, ethanol, butyrate/no	37–40	5.4–7.0	CO: 11.1, H_2_ + CO_2_: 25.0	CP009933	(Liou *et al*., 2005; Zhu *et al*., 2015)
*Desulfotomaculum thermobenzoicum subsp. thermosyntrophicum* DSM 14055	H_2_ + CO_2_	Acetate/no	55	7.0–7.5			(Plugge *et al*., 2002)
*Eubacterium aggregans* DSM 12183	H_2_ + CO_2_	Acetate, formate/yes	35	7.2		FNRK00000000	(Mechichi *et al*., 1998)
*Eubacterium limosum* DSM 20543	H_2_ + CO_2_, CO	Acetate, CO_2_/yes	39	7.0–7.2	CO: 7, H_2_ + CO_2_: 14	CP019962	(Eggerth, 1935; Bryant *et al*., 1958; Genthner *et al*., 1981; Genthner and Bryant, 1982 Song and Cho, 2015)
*Fuchsiella alkaliacetigena* DSM 24880	H_2_ + CO_2_	Acetate/no	40	8.8–9.3			(Zhilina *et al*., 2012)
*Fuchsiella ferrireducens* DSM 26031	H_2_ + CO_2_	Acetate/no	30–37	9.8			(Zhilina *et al*., 2015)
*Holophaga foetida* d DSM 6591	N.r.	N.r./no	28–32	6.8–7.5		AGSB02000000	(Liesack *et al*., 1994 Anderson *et al*., 2012)
*Marvinbryantia formatexigens* DSM 14469	H_2_ + CO_2_, formate	Acetate/no	37^b^	7.0^b^		ACCL00000000	(Wolin *et al*., 2003, 2008)
*Oxobacter pfennigii* DSM 3222	H_2_ + CO_2_, CO	Acetate, butyrate/no	36–38	7.3	CO: 13,9	LKET01000000	(Krumholz and Bryant, 1985; Bengelsdorf *et al*., 2015b)
*Sporomusa acidovorans* DSM 3132	H_2_ + CO_2_	Acetate/yes	35	6.5–7.0		LSLL00000000	(Ollivier *et al*., 1985 Humphreys *et al*., 2017a)
*Sporomusa aerivorans* DSM 13326	H_2_ + CO_2_	Acetate/yes	30	7.0	H_2_ + CO_2:_ 8.9		(Boga *et al*., 2003)
*Sporomusa malonica* DSM 5090	H_2_ + CO_2_	Acetate/yes	28–32	7.3		FWXI00000000	(Dehning *et al*., 1989)
*Sporomusa ovata* DSM 2662	H_2_ + CO_2_	Acetate/yes	34	6.3		ASXP01000008	(Möller *et al*., 1984 Poehlein *et al*., 2013)
*Sporomusa paucivorans* DSM 3697	H_2_ + CO_2_	Acetate/yes	34	6.7	H_2_ + CO_2:_ 10		(Hermann *et al*., 1987)
*Sporomusa rhizae* DSM 16652	H_2_ + CO_2_	Acetate/n.r.	35	7.5			(Gößner, 2006)
*Sporomusa silvacetica* DSM 10669	H_2_ + CO_2_	Acetate/yes	25–30	5.5–7.7		LSLK00000000	Kuhner *et al*., 1997 *;* Humphreys *et al*., 2017b)
*Sporomusa sphaeroides* DSM 2875	H_2_ + CO_2_	Acetate/yes	35–37	6.5		LSLJ00000000	(Möller *et al*., 1984 Castillo *et al*., 2017)
*Sporomusa termitida* DSM 4440	H_2_ + CO_2_, CO	Acetate/yes	30	7.2	H_2_ + CO_2:_ 7.8		(Breznak *et al*., 1988)
*Terrisporobacter glycolicus* RD‐1^a^ DSM 13865	H_2_ + CO_2_	Acetate/no	37–40	7.0–7.5		AUUB01000000	(Küsel *et al*., 2001; Gerritsen *et al*., 2014)
*Terrisporobacter mayombei* DSM 6539	H_2_ + CO_2_	Acetate/no	33	7.3	H_2_ + CO_2_: 5		(Kane *et al*., 1991; Gerritsen *et al*., 2014)
*Treponema primitia* DSM 12427	H_2_ + CO_2_	Acetate/no	30	7.2	H_2_ + CO_2_: 29	CP001843	(Graber *et al*., 2004; Graber and Breznak, 2004; Rosenthal *et al*., 2011)
*Calderihabitans maritimus* DSM 26464	CO	H_2_ + CO_2_ acetate/no	65	7.0–7.5		BDGJ00000000	(Yoneda *et al*., 2012; Omae *et al*., 2017)
*Carboxydothermus ferrireducens* DSM 11255	H_2_ + CO_2_, CO	N.r./no	65	6.0–6.2		ATYG00000000	Slobodkin *et al*., 1997; Slobodkin, 2006)
*Carboxydothermus hydrogenoformans* DSM 6008	CO	H_2_ + CO_2_/no	70–72	6.8–7.0	CO: 2	CP000141	(Svetlichny *et al*., 1991; Wu *et al*., 2005)
*Carboxydothermus pertinax* DSM 23698	H_2_ + CO_2_, CO	H_2_ + CO_2_/no	65	6.0–6.5	CO: 1.5	BDJK00000000	(Yoneda *et al*., 2012 *;*Fukuyama *et al*., 2017)
*Moorella glycerini* d DSM 11254	N.r.	Acetate/n.r.	58	6.3–6.5		CELZ00000000	(Slobodkin *et al*., 1997)
*Moorella mulderi* DSM 14980	H_2_ + CO_2_	Acetate/yes	65	7.0		LTBC00000000	(Balk *et al*., 2003; Castillo *et al*., 2016)
*Moorella thermoacetica* DSM 2955	H_2_ + CO_2_, CO	Acetate/yes	55	6.9	CO: 9‐16	CP012369	(Fontaine *et al*., 1942; Kerby and Zeikus, 1983; Andreesen *et al*., 1973; Daniel *et al*., 1990; Parekh and Cheryan, 1991; Gößner *et al*., 1999; Bengelsdorf *et al*., 2015a; Poehlein *et al*., 2015a)
*Moorella thermoautotrophica* DSM 1974	H_2_ + CO_2_, CO	Acetate/yes	56–60	5.7	H_2_ + CO_2_: 8		(Wiegel *et al*., 1981)

n.d., not deposited; n.r., not reported.

**a.** No type strain.

**b.** Condition not described as optimal, but used in the reference.

**c.** No validly described species.

**d.** No growth on gas reported, but all Wood–Ljungdahl pathway genes found in the genome.

*Clostridium ljungdahlii* has been isolated for its ability to grow on CO and CO‐containing gas mixtures such as syngas (Tanner *et al*., 1993). CO_2_/H_2_ gas mixtures cannot be utilized as efficient as by *A. woodii*. *C. ljungdahlii* also relies on a Rnf complex for generation of an ion gradient, but in this case, it is proton‐dependent (Tremblay *et al*., 2012) and thus coupled to a H^+^‐dependent ATPase. The organism is also genetically well accessible and completely sequenced. Recombinant strain construction (butanol) has been achieved as well (Köpke *et al*., 2010). *C. ljungdahlii* is meanwhile considered to be a model acetogen for proton bioenergetics and CO utilization.

A very close relative, *C. autoethanogenum*, was described only a few months after *C. ljungdahlii* (Abrini *et al*., 1994). Phylogenetically, it contains an identical 16S rRNA gene as *C. ljungdahlii* and both show a very high genome sequence similarity, however, no identity (> 98%; Humphreys *et al*., 2015). Its characteristics are very comparable to *C. ljungdahlii*, with only few differences (Humphreys *et al*., 2015). *C. autoethanogenum* was meanwhile developed into the industrial acetogen workhorse, being employed be the leading company in this field, LanzaTech, Inc (Skokie, IL, USA).

Finally, *M. thermoacetica* is a thermophilic acetogen, which was used as model organism for elucidation of the Wood–Ljungdahl pathway. Ironically, this was all performed with sugar‐grown cultures, as CO‐dependent growth was only found much later (Daniel *et al*., 1990). Besides being the best characterized acetogenic thermophile, *M. thermoacetica* also uses a different bioenergetic system. The organism possesses an Ech complex (no Rnf) and, in addition, also cytochromes and menaquinone (Gottwald *et al*., 1975; Das and Ljungdahl, 2003; Pierce *et al*., 2008; Schuchmann and Müller, 2014). Thus, there are two potential possibilities to generate a proton gradient across the membrane.

## Aerobic metabolism of CO, H_2_ and CO_2_ containing gases

Whereas anaerobic fermentation of CO, H_2_ and CO_2_‐containing gases with acetogenic bacteria is well known and already employed for industrial ethanol production, the aerobic utilization of such gases for biotechnological purposes is still in its infancy and has not been exploited so far. This certainly is due to the fact that in spite of numerous organisms able to grow chemolithoautotrophically on H_2_, CO_2_ and CO mixtures (King, 2003; King and Weber, 2007), only few of these so‐called carboxydotrophic bacteria have been characterized in detail, molecular toolboxes for these organisms have not been developed, H_2_ and CO show very low solubility in aqueous solutions, CO is highly toxic, and the handling of H_2_ and CO in the presence of O_2_ requires extensive precautions. However, aerobic CO oxidation is energetically more favourable than anaerobic oxidation and subsequent acetogenesis (reactions 1 and 2, respectively; Diender *et al*., 2015) and, thus, aerobic oxidation of CO should allow the production of more costly (i.e. ATP‐intensive) products than CO oxidation via anaerobic acetogenesis.

$$\text{Reaction I}:2\;\text{CO}+O_{2}\to 2\;{\text{CO}}_{2}\;\;\left(\Delta G^{0}=-514\;\text{kJ}\right)$$

$$\begin{array}{cc} & \text{Reaction II}:4\;\text{CO}+2{\text{H}}_{2}\;\text{O}\to {\text{CH}}_{3}{\text{COO}}^{-}+{\text{H}}^{+}+2\;{\text{CO}}_{2}\\ & \;\;\;\;\;\;\;\;(\Delta {\text{G}}^{0}=-174\;\text{kJ}) \end{array}$$

Accordingly, bioreactors for aerobic, carboxydotrophic cultivation need to be equipped with sufficient cooling capacities to buffer the heat release caused by necessarily tightly controlled H_2_ oxidation ($\Delta H_{C}^{0}$ = 286 kJ mole^−1^) which somewhat resembles the cooling demands of comparable sugar‐based, aerobic scenarios.

Species of *Oligotropha, Bradyrhizobium, Mesorhizobium, Hydrogenophaga, Burkholderia* and also some species of *Mycobacterium*,* Pseudomonas, Alcaligenes* and *Acinetobacter* have been reported to grow aerobically on CO and CO‐ and H_2_‐containing gases as sole carbon and energy sources (reviewed in Meyer and Schlegel, 1983; King, 2003; King and Weber, 2007; Weber and King, 2012). Especially, carboxydotrophic bacteria such as *Oligotropha carboxidovorans* and *Hydrogenophaga pseudoflava* which possess a highly CO‐tolerant respiratory chain and show high growth rates under autotrophic conditions are promising candidates for future biotechnological application in aerobic gas fermentations (Zavarzin and Nozhevnikova, 1977; Cypionka *et al*., 1980; Cypionka and Meyer, 1982). *Oligotropha carboxidovorans* probably is the best studied carboxydotrophic bacterium. It possesses a CO‐insensitive aerobic electron transport chain, an O_2_‐tolerant molybdenum and copper‐containing CO dehydrogenase for oxidation of CO_2_ and the Calvin–Benson–Bassham cycle for fixation of CO_2_ during autotrophic growth on CO and CO_2_ (Meyer and Schlegel, 1978, 1983). *O. carboxidovorans* is also able to grow organoheterotrophically with organic acids (Meyer and Schlegel, 1983), its genome consists of one chromosome and two megaplasmids, one of which (pHCG3) harbours the (substrate‐inducible) genes required for H_2_ and CO oxidation and for CO_2_ fixation (Fuhrmann *et al*., 2003; Paul *et al*., 2008; Volland *et al*., 2011). However, genetic tools are so far not available for this organism.

Very recently, Heinrich *et al*. (2017) reported on aerobic utilization of syngas by recombinant strains of *Ralstonia eutropha* H16 (currently designated as *Cupriavidus necator* H16). The wild type of this species is an aerobic and chemolithoautotrophic ‘Knallgas’ bacterium able to efficiently use H_2_ and CO_2_ as sole carbon and energy sources and possessing a hydrogenase and an electron chain which are relatively insensitive towards CO (Cypionka and Meyer, 1982; Friedrich and Schwartz, 1993; Bürstel *et al*., 2016). *C. necator* H16 was genetically engineered to express the genes encoding the *O. carboxidovorans* CO dehydrogenase as well as the genes encoding proteins for maturation of this enzyme. In the presence of H_2_, CO_2_ and CO (plus small amounts of a heterotrophic substrate in the preculture), the resulting strain was able to (slowly) oxidize and use CO as a carbon source, grew slightly faster and produced significantly more poly‐D‐3‐hydroxybutyrate (PHB) than the parental strain carrying the empty plasmid on H_2_ and CO_2_ (Heinrich *et al*., 2017). Since *R. eutropha* has been shown to produce several recombinant higher‐value products aside from PHB (reviewed in Dürre and Eikmanns, 2015), the metabolic engineering strategy applied seems very promising for further biotechnological exploitation.

The development of genetic tools for aerobic, carboxydotrophic bacteria is still in the beginning. However, genome analysis of promising representatives, establishment of sophisticated genetic engineering tools such as efficient transformation, differential expression of homologous and heterologous genes, markerless deletions and/or allelic exchanges should allow the construction of one or more carboxydotrophic (platform or model) organism/s for future biotechnological applications, that is for aerobic utilization of syngas for the production of value‐added products.

## Bioprocess developments

Autotrophic growth of acetogens is extremely energy limited and gas–liquid mass transport limitations restrict biocatalytic activities due to the low solubilities of the gaseous substrates H_2_ and CO in water at ambient pressure. H_2_ is 65 mol% and CO is 75 mol% less soluble in water compared to O_2_ (1 bar, 20 °C). Compared to typical aerobic heterotrophic bioprocesses, planktonic cell concentrations are reduced by a factor of 10 and more, resulting in low volumetric productivities of gas fermentations.

Gas–liquid mass transfer can be improved by increasing the volumetric power input into bioreactors with dispersed gas phase (e.g. increasing the stirrer speed in stirred‐tank bioreactors) and/or increasing the partial pressures of H_2_ and CO in the gas phase. Increasing the power input per unit volume will cause severe scale‐up challenges and will be economically demanding if low value adding products are produced from syngas like C2‐C4 alcohols or acids. Increasing the partial pressures of H_2_ and CO will thus be the method of choice, for example by applying bioreactors with liquid heights *h* of 20–30 m to build up a high hydrostatic pressure at the bottom of the reactor where the syngas is dispersed. Amongst others, these are the reasons why bubble‐column or gas‐lift reactors are chosen for syngas fermentations with planktonic cells on an industrial scale. It must be pointed out that increased partial pressures of the gaseous substrates are favourable from a thermodynamic point of view as well.

The power input of bubble‐column reactors on an industrial scale is caused by the isothermal expansion of the gas phase dispersed at the bottom of the reactor (*P*_Exp_)

(1) $$P_{\mathrm{Exp}}=\frac{{\dot{V}}_{\mathrm{gas}}\cdot \rho_{\mathrm{gas}}}{{\tilde{M}}_{\mathrm{gas}}}\cdot \text{RT}\cdot \text{In}\left(\frac{1+\rho_{L}\cdot g\cdot h}{p_{U}}\right),$$

with ${\dot{V}}_{\mathrm{gas}}$ as the inlet gas flow rate, *ρ*_gas_ as the density of the gas, ${\tilde{M}}_{\mathrm{gas}}$ as the molecular weight of the gas, *p*_*U*_ as the pressure above the liquid surface (head‐space pressure), *ρ*_L_ as the density of the liquid phase, *g* as the acceleration of gravity and *h* as the liquid height above the gas sparger in the reactor. The power input *P*_Exp_ increases with raising gas inlet flow rate and liquid height *h*. Due to the elevated partial pressures of the gases in the inlet at high liquid heights, increasing the liquid height *h* results in elevated partial pressures at the bottom of the bubble‐column reactor and increased power input both serving for improved gas–liquid mass transfer. As a consequence, solely acetogenic producer strains can be applied for syngas fermentation which are not inhibited by increased H_2_‐ or CO‐partial pressures of up to a few bar. Unfortunately, H_2_‐ or CO‐inhibition kinetics of acetogens are not very well studied so far (e.g. Vega *et al*., 1988; Chang *et al*., 1998; Skidmore *et al*., 2013; Mohammadi *et al*., 2014).

The partial pressures in the gas bubbles rising in a bubble column vary considerably as function of the height *h* in the column due to the consumption and production of gases by acetogens in the liquid phase and due to the decline of the total pressure. As a consequence, axial gradients are inevitable with respect to the partial pressures of the gas phase as well as the concentrations of biomass, products and pH in the liquid phase along the height of the bubble‐column reactor. Whereas the multiphase transport processes can be described by well‐known modelling approaches (e.g. plug flow of both phases with axial dispersion and gas–liquid mass transport in bubble columns), the gas consumption and product formation kinetics of acetogens as function of substrate (H_2_, CO) and product concentrations (acetate, ethanol, etc.) as well as pH are not very well known. An approach to overcome this lack of kinetic information is making use of a genome‐scale metabolic reconstruction of acetogens combined with uptake kinetics for H_2_ and CO (e.g. Chen *et al*., 2015).

Further studies on the kinetics of acetogens in fully controlled and well‐mixed laboratory‐scale stirred‐tank bioreactors are inevitable to provide the kinetic data which are needed for the modelling of syngas fermentation in bubble columns on an industrial scale. Batch syngas fermentation processes in stirred‐tank bioreactors will provide basic performance data (e.g. Demler and Weuster‐Botz, 2011, Groher and Weuster‐Botz, 2016a,b; Kantzow and Weuster‐Botz, 2016; Mayer and Weuster‐Botz, 2017) but continuous syngas fermentations should be preferred due to the possibility to perform steady‐state studies (e.g. Mohammadi *et al*., 2012). Low growth rates of acetogens especially if extreme reaction conditions are to be studied may be an obstacle (e.g. pH, T, inhibiting gas concentrations). The application of submerged microfiltration membranes in a continuously operated stirred‐tank bioreactor enables the study of syngas fermentations with (total) cell retention (Kantzow *et al*., 2015). A cascade of stirred‐tank bioreactors is another option with the first reactor operated at optimum autotrophic growth conditions for acetogens and the second reactor at extreme reaction conditions, for example for studying the reconsumption of acids and the production of alcohols at low pH without growth (Richter *et al*., 2013; Martin *et al*., 2015).

One of the first commercial plants for the conversion of CO‐rich industrial off‐gases of a steel mill is presently under construction at the ArcelorMittal steel mill in Ghent (Belgium) making use of continuously operated bubble‐column/gas‐lift loop reactors. The syngas fermentation process developed by LanzaTech uses *Clostridium autoethanogenum* for the production of ethanol with an estimated volumetric productivity of around 10 g l^−1^ h^−1^. The syngas fermentation plant in Ghent is designed for an annual production capacity of 60.000 m^3^ ethanol converting 50.000 Nm^3^ h^−1^ steel mill off‐gases (Molitor *et al*., 2016) and is due to be operational in 2019. CO utilization of around 70–75% was shown at the Shougang (China) demonstration facility of LanzaTech (Heijstra *et al*., 2017). Current approaches even strive for almost complete CO utilization.

A promising alternative to bubble columns or gas‐lift reactors may be trickle‐bed biofilm reactors. A biofilm is formed on the (inner) surfaces of carrier materials forming a fixed‐bed in a cylindrical reactor. The liquid phase is distributed above the fixed‐bed and trickles down forming a thin liquid film on the surface of the carriers. In contrast to bubble‐column reactors, the gas phase is not dispersed in the liquid phase but forms the continuous phase in the trickle‐bed reactor. The pressure drop of the gas phase is thus negligible, and the power input of trickle‐bed reactors is solely caused by pumping the liquid phase to the top of the fixed‐bed of the trickle‐bed reactor. Despite the low power input, the gas–liquid and gas‐biofilm mass transfer becomes high at low liquid film thickness, high flow rates of the trickling liquid and low biofilm thickness. Due to the low operating costs at high mass transfer rates, trickle‐bed biofilm reactors and a special design of thereof, so‐called horizontal rotating packed‐bed biofilm reactors, are widespreadly used in waste water treatment plants.

Very few studies have been reported so far on the application of trickle‐bed reactors for syngas fermentation on a laboratory‐scale (e.g. Bredwell *et al*., 1999; Yasin *et al*., 2015; Devarapalli *et al*., 2016; Schulte *et al*., 2016; Shen *et al*., 2017). High CO conversion rates of up to 91% were observed with *Clostridium ragsdalei* in a trickle‐bed reactor with non‐porous glass beads of 6 mm as carriers (Devarapalli *et al*., 2016). H_2_ utilization of more than 80% was measured in a laboratory‐scale horizontal rotating packed‐bed biofilm reactor with *Clostridium carboxidivorans* P7 with non‐porous HDPE carriers with a specific surface area of 500 m^2^ m^‐3^. Compared to the syngas fermentation with a continuous operated stirred‐tank bioreactor at the same operation conditions, the volumetric ethanol productivity was 3.3 times higher (Shen *et al*., 2017). Biofilm formation seems to be not an issue with acetogens although systematic studies are missing so far.

Axial gradients of pH and product concentrations are inevitable in the trickling liquid phase along the height of a trickle‐bed reactor on an industrial scale (10 ‐ 15 m). Recycling of the liquid phase may be a solution to reduce gradients but will increase the volumetric power input. Compared to bubble‐column or gas‐lift reactors, the liquid phase and biomass volume (‘working volume’) are very much reduced in trickle‐bed reactors to about 20% of the total volume compared to about 80% in bubble‐column reactors. The reduced working volume of trickle‐bed biofilm reactors will be balanced by improved mass transfer which is shown by higher volumetric productivities. However, industrial application of trickle‐bed biofilm reactors for syngas fermentation will only be possible if a stable biofilm can be established for long‐term operation, especially if recombinant acetogens will be applied for the (improved) production of natural or non‐natural products. Unfortunately, no studies on control, stability and long‐term operation of biofilms with (recombinant) acetogens have been published so far.

## Downstream processing

**Figure 3:**
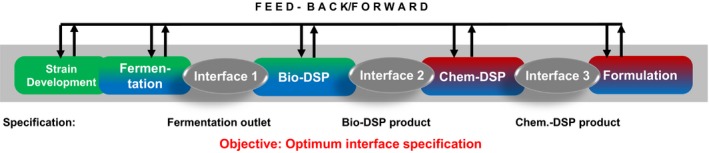
Bioprocess development to replace intermediates of an existing VAC by bioproducts. The need to fulfil given specifications but also to check for distinct bio‐originated new ones is outlined by the feed forward and backward loops.

‘Molecules don't jump out of the broth’. The old wisdom is still valid, for commodities as well as for performance molecules. Given that 30–50% of manufacturing costs of commodities are typically assigned to downstream processing (DSP), processes may gain or lose their economic viability in the downstream section. In general, DSP aims at providing products that meet given purity specifications. However, specification sheets of VAC intermediates and other commoditized molecules are mainly derived from fossil‐based production. Accordingly, listed impurities mirror the needs when dealing with fossil raw materials and do not cover impurity patterns of bioprocesses. A basic change of mindset is necessary, because fossil‐based impurities enter processes as co‐substrates while bioprocesses typically produce non‐wanted impurities via metabolic reactions. In consequence, specifications need to be revisited to fulfil their purpose of guaranteeing the performance of the respective product within the VAC and for the application. By‐products of fermentation need to be integrated in ‘conventional’ specification lists checking whether or not their occurrence hampers the efficiency of subsequent processing steps or even the functionality of the final product. For instance, low impurity concentrations may cause non‐wanted caking in DSP which in turn may necessitate repeated strain engineering to prevent by‐product formation. The example outlines the crucial importance of mass balancing in DSP to track impurities. Furthermore, it shows that total bioprocess development is a workflow of different activities with distinct interfaces and feedback loops that needs to be cycled several times to succeed (Fig. 3).

## Intrinsic benefits of gas fermentation evaluated by conceptual design

As outlined above, the minimization of manufacturing costs is key for the production of VAC intermediates and commodities. Accordingly, criteria shown in Figure 1 need to be optimized. Some helpful evaluation may even be performed during early‐stage conceptual design using rather simple but characteristic assumptions. Considering a common sugar‐based production capacity of 100 kilo‐tons per annum (kta), average space–time yield (STY) of 4 g l^−1^ h^−1^, product titre of 100 g^−^l^−1^ (achieved after 40 h batch‐time including turnaround), total product recovery of 85% and operation time of 8000 h a^−1^, the following conclusion can be drawn: 200 batch cycles per annum will be necessary, cycling about 5882 m³ batch^−1^. Consequently, 12 bioreactors, each ≥ 500 m³ working volume will be needed. However, gas fermentations offer the advantage to run in continuous mode which will extend ‘batch‐times’ to 100 h. As a result, only 6 bioreactors≥ 500 m³ working volume will be needed which illustrates the economic potential to save CAPEX (capital expenditure) and OPEX (operational expenditure) with gas fermentations. To improve the latter, gas fermentations typically consider operational pressures up to 4 bar for ensuring sufficient mass transfer and reasonable specific power input <0.3 kW m^−^³ in combination with loops to consume the gaseous substrates as good as possible.

## Size matters – large‐scale production of intermediates

Based on decades of experience within chemical process engineering, routines and short cut methods exist to predict large‐scale capital and operational expenditures. Such algorithms typically predict specific CAPEX decrease with increasing capacity because costs of apparatuses nonlinearly increase with size. Beyond maximum apparatus size, numbering‐up of equal devices is applied which keeps specific CAPEX constant. Such limits strongly depend on the devices used. For instance, solid‐/liquid separation equipment such as centrifuges reaches the limit of scale‐up much earlier than liquid processing equipment such as distillation columns.

The principle also holds true for different types of reactors. Continuous chemical reactor systems usually reach their maximum economy of scale at significantly higher capacities than batch‐type bioreactors. Of course, this depends on process‐specific performance indicators, that is space–time yield and catalyst‐specific productivity. Accordingly, a fossil‐based process to produce a commodity such as acrylic acid might reach its maximum economy of scale at capacities > 200 kt a^−1^. In comparison, the fermentative production analogue of the respective precursor will get at the said limit already < 100 kt a^−1^. In other words, bioprocesses accomplish economy of scale with less product than chemical counterparts.

The relation between CAPEX of a specific intermediate and the capacity of a respective bio‐based process and production plant significantly depends on the ‘nature’ of the product and in consequence the efforts to be taken to deplete impurities. The lowest cost might be achieved with a low‐boiling product that can be purified by distillation of the aqueous reaction mixture, for example ethanol. Also, gaseous products are advantageous in terms of DSP in case those could be purified via fractional condensation or pressure swing adsorption. Products which need to be handled as crystals demand higher specific CAPEX since solid‐/liquid separation and solids processing apparatus come at higher specific cost.

## The perspective

As already outlined, gas fermentations using mixtures of CO, CO_2_ and H_2_ possess the inherent potential to substitute fossil‐derived components by bio‐based intermediates thereby continuing with already established VACs. CAPEX and OPEX scenarios look promising for production capacities > 100 kt a^−1^ provided that product specifications are met. The successful solution of this challenge asks for improvements in strain and process engineering. Intrinsic problems of mass transfer and bioreactor design need to be solved which demands for the application of novel knowledge‐based scale‐up approach as already outlined in Takors (2012) and Delvigne *et al*. (2017). Besides, the economic access to electron‐donating substrates (CO, H_2_) is a prerequisite of every industrial process. Individual solutions may be found in highly networked composite chemical sites or via integration in future international energy grids.

CO_2_, CO and H_2_ were in the focus of this review, but alternatives such as CH_4_ may be promising as well. The latter offers access to ATP‐demanding product biosynthesis under aerobic conditions, thereby bypassing the intrinsic ATP limitations of anaerobic CO_2_, CO and H_2_ metabolism. However, aerobic carboxydotrophic strains may be another alternative requiring intensified research and sensitive large‐scale engineering in compliance with ATEX regulations (ATmosphères EXplosibles).

Currently, companies such as LanzaTech are succeeding to implement industrial scale gas fermentation for the production of native products such as ethanol or 2,3‐butanediol. However, the turnaround from fossil raw materials to zero‐CO_2_ emission requires for much more VAC intermediates, as outlined above. The time is right to start research.

## Conflict of interest

None declared.

## References

[mbt213270-bib-0001] Abrini, J. , Naveau, H. , and Nyns, E. (1994) *Clostridium autoethanogenum*, sp. nov., an anaerobic bacterium that produces ethanol from carbon monoxide. Arch Microbiol 161: 345–351. doi:10.1007/s00203005006

[mbt213270-bib-0002] Abubackar, H.N. , Veiga, M.C. , and Kennes, C. (2011) Biological conversion of carbon monoxide: rich syngas or waste gases to bioethanol. Biofuels Bioprod Bioref 5: 93–114.

[mbt213270-bib-0003] Allen, T.D. , Caldwell, M.E. , Lawson, P.A. , Huhnke, R.L. , and Tanner, R.S. (2010) *Alkalibaculum bacchi* gen. nov., sp. nov., a CO‐oxidizing, ethanol‐producing acetogen isolated from livestock‐impacted soil. Int J Syst Evol Microbiol 60: 2483–2489. https://doi.org/10.1099/ijs.0.018507-0 19965999 10.1099/ijs.0.018507-0

[mbt213270-bib-0500] Andreesen, J.R. , Schaupp, A. , Neurauter, C. , Brown, A. , and Ljungdahl, L.G. (1973) Fermentation of glucose, fructose, and xylose by Clostridium thermoaceticum: Effect of metals on growth yield, enzymes, and the synthesis of acetate from CO_2_ . J Bacteriol 114: 743–751. 4706193 10.1128/jb.114.2.743-751.1973 PMC251835

[mbt213270-bib-0004] Anderson, I. , Held, B. , Lapidus, A. , Nolan, M. , Lucas, S. , Tice, H. , *et al* (2012) Genome sequence of the homoacetogenic bacterium Holophaga foetida type strain (TMBS4T) Stand. Genomic Sci 6, 174–184. https://doi.org/10.4056/sigs.2746047 10.4056/sigs.2746047 PMC3387795 22768361

[mbt213270-bib-0005] Andreesen, J.R. , Gottschalk, G. , and Schlegel, H.G. (1970) *Clostridium formicoaceticum* nov. spec. Isolation, description and distinction from *C. aceticum* and *C. thermoaceticum* . Arch Mikrobiol 72: 154–174. https://doi.org/10.1007/BF00409521 4918913 10.1007/BF00409521

[mbt213270-bib-0006] Arnold, S. , Moss, K. , Henkel, M. , and Hausmann, R. (2017) Biotechnological perspectives of pyrolysis oil for a bio‐based economy. Trends Biotechnol 35: 925–936. https://doi.org/10.1016/j.tibtech.2017.06.003 28666545 10.1016/j.tibtech.2017.06.003

[mbt213270-bib-0501] Bache, R. , and Pfennig, N. (1981) Selective isolation of Acetobacterium woodii on methoxylated aromatic acids and determination of growth yields. Arch Microbiol 130: 255–261.

[mbt213270-bib-0502] Balk, M. , Weijma, J. , Friedrich, M.W. , and Stams, A.J. (2003) Methanol utilization by a novel thermophilic homoacetogenic bacterium, Moorella mulderi sp. nov., isolated from a bioreactor. Arch Microbiol 179: 315–320. 12637975 10.1007/s00203-003-0523-x

[mbt213270-bib-0007] Balch, W.E. , Schoberth, S. , Tanner, R.S. , and Wolfe, R.S. (1977) *Acetobacterium*, a new genus of hydrogen‐oxidizing, carbon dioxide‐reducing, anaerobic bacteria. Int J Syst Bacteriol 27: 355–361. https://doi.org/10.1099/00207713-27-4-355

[mbt213270-bib-0008] Bengelsdorf, F.R. , and Dürre, P. (2017) Gas fermentation for commodity chemicals and fuels. Microb Biotechnol 10: 1167–1170. https://doi.org/10.1111/1751-7915.12763 28696068 10.1111/1751-7915.12763 PMC5609230

[mbt213270-bib-0009] Bengelsdorf, F.R. , Poehlein, A. , Esser, C. , Schiel‐Bengelsdorf, B. , Daniel, R. , and Dürre, P. (2015a) Complete genome sequence of the acetogenic bacterium *Moorella thermoacetica* DSM 2955^T^ . Genome Announc 3: e01157–15. https://doi.org/10.1128/genomea.01157-15 26450730 10.1128/genomeA.01157-15 PMC4599089

[mbt213270-bib-0010] Bengelsdorf, F.R. , Poehlein, A. , Schiel‐Bengelsdorf, B. , Daniel, R. , and Dürre, P. (2015b) Genome sequence of the acetogenic bacterium *Oxobacter pfennigii* DSM 3222^T^ . Genome Announc 3: e01408–e01415. https://doi.org/10.1128/genomea.01408-15 10.1128/genomeA.01408-15 PMC4669397 26634756

[mbt213270-bib-0011] Bengelsdorf, F.R. , Poehlein, A. , Linder, S. , Erz, C. , Hummel, T. , Hoffmeister, S. , *et al* (2016a) Industrial acetogenic biocatalysts: a comparative metabolic and genomic analysis. Front Microbiol 7: 1036 https://doi.org/10.3389/fmicb.2016.01036 27458439 10.3389/fmicb.2016.01036 PMC4935695

[mbt213270-bib-0012] Bengelsdorf, F.R. , Poehlein, A. , Schiel‐Bengelsdorf, B. , Daniel, R. , and Dürre, P. (2016b) Genome sequence of the acetogenic bacterium *Butyribacterium methylotrophicum* DSM 3468. Genome Announc 4: e01338–16. https://doi.org/10.1128/genomea.01338-16 27908997 10.1128/genomeA.01338-16 PMC5137411

[mbt213270-bib-0013] Bengelsdorf, F.R. , Beck, B.H. , Erz, C. , Hoffmeister, S. , Karl, M.M. , Riegler, P. , *et al* (2018) Bacterial anaerobic synthesis gas (syngas) and CO_2_ + H_2_ fermentation. Adv Appl Microbiol 101, 2619–2627. 10.1016/bs.aambs.2018.01.002 29914657

[mbt213270-bib-0014] Bernalier, A. , Willems, A. , Leclerc, M. , Rochet, V. , and Collins, M.D. (1996) *Ruminococcus hydrogenotrophicus* sp. nov., a new H_2_/CO_2_‐utilizing acetogenic bacterium isolated from human feces. Arch Microbiol 166: 176–183. https://doi.org/10.1007/s002030050373 8703194 10.1007/s002030050373

[mbt213270-bib-0015] Bertuccioli, L. , Chan, A. , Hart, D. , Lehner, F. , Madden, B. and Standen, E. (2014) Development of water electrolysis in the European Union. Fuel cells and hydrogen joint undertaking 83: 1–160.

[mbt213270-bib-0016] Biegel, E. , and Müller, V. (2010) Bacterial Na^+^‐translocating ferredoxin: NAD^+^ oxidoreductase. Proc Natl Acad Sci USA 107: 18138–18142. https://doi.org/10.1073/pnas.1010318107 20921383 10.1073/pnas.1010318107 PMC2964206

[mbt213270-bib-0017] Boga, H.I. , Ludwig, W. , and Brune, A. (2003) Sporomusa aerivorans sp. nov., an oxygenreducing homoacetogenic bacterium from the gut of a soil feeding termite. Int J Syst Evol Microbiol 53: 1397–1404. 13130024 10.1099/ijs.0.02534-0

[mbt213270-bib-0506] Bomar, M. , Hippe, H. , and Schink, B. (1991) Lithotrophic growth and hydrogen metabolism by Clostridium magnum. FEMS Microbiol Let 83: 347–350. 10.1016/0378-1097(91)90500-a 1769543

[mbt213270-bib-0018] Braun, M. , and Gottschalk, G. (1982) *Acetobacterium wieringae* sp. nov., a new species producing acetic acid from molecular hydrogen and carbon dioxide. Zentralbl Bakteriol Mikrobiol Hyg. I. Abt. 3: 368–376. https://doi.org/10.1016/s0721-9571(82)80017-3

[mbt213270-bib-0019] Braun, M. , Mayer, F. , and Gottschalk, G. (1981) *Clostridium aceticum* (Wieringa), a microorganism producing acetic acid from molecular hydrogen and carbon dioxide. Arch Microbiol 128: 288–293. https://doi.org/10.1007/BF00422532 6783001 10.1007/BF00422532

[mbt213270-bib-0020] Bredwell, M.D. , Srivastava, P. , and Worden, R.M. (1999) Reactor design issues for synthesis gas fermentations. Biotechnol Prog 15: 834–844. 10514253 10.1021/bp990108m

[mbt213270-bib-0021] Breznak, J.A. , Switzer, J.M. , and Seitz, H. (1988) *Sporomusa termitida* sp. nov., an H_2_/CO_2_‐utilizing acetogen isolated from termites. Arch Microbiol 150: 282–288. https://doi.org/10.1007/BF00407793

[mbt213270-bib-0022] Brown, S.D. , Nagaraju, S. , Utturkar, S. , Tissera, S.D. , Segovia, S. , Mitchell, W. , *et al* (2014) Comparison of single‐molecule sequencing and hybrid approaches for finishing the genome of *Clostridium autoethanogenum* and analysis of CRISPR systems in industrial relevant Clostridia. Biotechnol Biofuels 7: 40 https://doi.org/10.1186/1754-6834-7-40 24655715 10.1186/1754-6834-7-40 PMC4022347

[mbt213270-bib-0023] Bryant, M. , Small, N. , Bouma, C. , and Robinson, I. (1958) Studies on the composition of the ruminal flora and fauna of young calves. J Dairy Sci 41: 1747–1767. https://doi.org/10.3168/jds.S0022-0302(58)91160-3

[mbt213270-bib-0024] Bürstel, I. , Siebert, E. , Frielingsdorf, S. , Zebger, I. , Friedrich, B. , and Lenz, O. (2016) CO synthesized from the central one‐carbon pool as source for the iron carbonyl in O_2_‐tolerant [NiFe]‐hydrogenase. Proc Natl Acad Sci USA 113: 14722–14726. 27930319 10.1073/pnas.1614656113 PMC5187695

[mbt213270-bib-0504] Castillo Villamizar, G.A. , and Poehlein, A. (2016) Genome sequence of the acetogenic bacterium *Moorella mulderi* DSM 14980^T^ . Genome Announc 4: e00444–16. 27231372 10.1128/genomeA.00444-16 PMC4882953

[mbt213270-bib-0505] Castillo Villamizar, G.A. , Daniel, R. , and Poehlein, A. (2017) First insights into the genome sequence of the strictly anaerobic homoacetogenic *Sporomusa sphaeroides* strain E (DSM 2875). Genome Announce 5: e00037–17. 10.1128/genomeA.00037-17 PMC5364215 28336590

[mbt213270-bib-0025] Chang, I.‐S. , Kim, D.‐H. , Kim, B.‐H. , Shin, P.‐K. , Sung, H.‐C. , and Lovitt, R.W. (1998) CO fermentation of *Eubacterium limosum* KIST612. J Microbiol Biotechnol 8: 134–140.

[mbt213270-bib-0026] Chen, S. , Beeby, M. , Murphy, G.E. , Leadbetter, J.R. , Hendrixson, D.R. , Briegel, A. , *et al* (2011) Structural diversity of bacterial flagellar motors. EMBO J 30: 2972–2981. https://doi.org/10.1038/emboj.2011.186 21673657 10.1038/emboj.2011.186 PMC3160247

[mbt213270-bib-0027] Chen, J. , Gomez, J.A. , Höffner, K. , Barton, P.I. , and Henson, M.A. (2015) Metabolic modeling of synthesis gas fermentation in bubble column reactors. Biotechnol Biofuels 8: 89. 26106448 10.1186/s13068-015-0272-5 PMC4477499

[mbt213270-bib-0028] Cypionka, H. , and Meyer, O. (1982) Influence of carbon monoxide on growth and respiration of carboxydobacteria and other aerobic organisms. FEMS Microbiol Lett 15: 209–214.

[mbt213270-bib-0029] Cypionka, H. , Meyer, O. , and Schlegel, H.G. (1980) Physiological characteristics of various species of strains of carboxydobacteria. Arch Microbiol 127: 301–307.

[mbt213270-bib-0030] Daniel, S.L. , Hsu, T. , Dean, S.I. , and Drake, H.L. (1990) Characterization of the H_2_‐ and CO‐dependent chemolithotrophic potentials of the acetogens *Clostridium thermoaceticum* and *Acetogenium kivui* . J Bacteriol 172: 4464–4471. https://doi.org/10.1128/jb.172.8.4464-4471.1990 2376565 10.1128/jb.172.8.4464-4471.1990 PMC213276

[mbt213270-bib-0031] Daniell, J. , Köpke, M. , and Simpson, S.D. (2012) Commercial biomass syngas fermentation. Energies 5: 5372–5417.

[mbt213270-bib-0032] Das, A. , and Ljungdahl, L.G. (2003) Electron‐transport systems in acetogens In Biochemistry and physiology of anaerobic bacteria. Ljungdahl L.G., Adams M.W., Barton L.L., Ferry J.G., and Johnson M.K. (eds). New York, NY: Springer Verlag, pp. 191–204.

[mbt213270-bib-0033] Dehning, I. , Stieb, M. , and Schink, B. (1989) *Sporomusa malonica* sp. nov., a homoacetogenic bacterium growing by decarboxylation of malonate or succinate. Arch Microbiol 151: 421–426. https://doi.org/10.1007/BF00416601

[mbt213270-bib-0034] Delvigne, F. , Takors, R. , Mudde, R. , van Gulik, W. , and Noorman, H. (2017) Bioprocess scale‐up/down as integrative enabling technology: from fluid mechanics to systems biology and beyond. Microb Biotechnol 10: 1267–1274. https://doi.org/10.1111/1751-7915.12803 28805306 10.1111/1751-7915.12803 PMC5609235

[mbt213270-bib-0035] Demler, M. , and Weuster‐Botz, D. (2011) Reaction engineering analysis of hydrogenotrophic production of acetic acid by *Acetobacterium woodii* . Biotechnol Bioeng 108: 470–474. 20830677 10.1002/bit.22935

[mbt213270-bib-0036] Devarapalli, M. , Atiyeh, H.K. , Phillips, J.R. , Lewis, R.S. , and Huhnke, R.L. (2016) Ethanol production during semi‐continuous syngas fermentation in a trickle bed reactor using *C. ragsdalei* . Biores Technol 209: 56–65. https://doi.org/10.1016/j.biortech.2016.02.086 10.1016/j.biortech.2016.02.086 26950756

[mbt213270-bib-0037] Diender, M. , Stams, A.J.M. , and Sousa, D.Z. (2015) Pathways and bioenergetics of anaerobic carbon monoxide fermentation. Front Microbiol 6: 1275. 26635746 10.3389/fmicb.2015.01275 PMC4652020

[mbt213270-bib-0038] Dürre, P. (2016) Gas fermentation – a biotechnological solution for today's challenges. Microbial Biotechnol 10: 14–16. 10.1111/1751-7915.12431 PMC5270713 27790842

[mbt213270-bib-0039] Dürre, P. , and Eikmanns, B.J. (2015) C1‐carbon sources for chemical and fuel production by microbial gas fermentation. Curr Opin Biotechnol 35: 63–72. 25841103 10.1016/j.copbio.2015.03.008

[mbt213270-bib-0040] Eggerth, A.H. (1935) The Gram‐positive non‐spore‐bearing anaerobic bacilli of human feces. J Bacteriol 30: 277–299. 16559837 10.1128/jb.30.3.277-299.1935 PMC543656

[mbt213270-bib-0041] Eichler, B. , and Schink, B. (1984) Oxidation of primary aliphatic alcohols by *Acetobacterium carbinolicum* sp. nov., a homoacetogenic anaerobe. Arch Microbiol 140: 147–152. https://doi.org/10.1007/BF00454917

[mbt213270-bib-0605] Fontaine, F.E. , Peterson, W.H. , McCoy, E. , Johnson, M.J. , and Ritter, G.J. (1942) A new type of glucose fermentation by *Clostridium thermoaceticum* . J Bacteriol 43: 701. 16560531 10.1128/jb.43.6.701-715.1942 PMC373636

[mbt213270-bib-0042] Friedrich, B. , and Schwartz, E. (1993) Molecular biology of hydrogen utilization in aerobic chemolithotrophs. Annu Rev Microbiol 47: 351–383. 8257102 10.1146/annurev.mi.47.100193.002031

[mbt213270-bib-0043] Fuhrmann, S. , Ferner, M. , Jeffke, T. , Henne, A. , Gottschalk, G. , and Meyer, O. (2003) Complete nucleotide sequence of the circular megaplasmid pHCG3 of *Oligotropha carboxidovorans*: function in the chemolithoautotrophic utilization of CO, H_2_ and CO_2_ . Gene 322: 67–75. 14644498 10.1016/j.gene.2003.08.027

[mbt213270-bib-0606] Fukuyama, Y. , Omae, K. , Yoneda, Y. , Yoshida, T. , and Sako, Y. (2017) Draft genome sequences of *Carboxydothermus pertinax* and *C. islandicus*, hydrogenogenic carboxydotrophic bacteria. Genome Announc 5: e01648‐16. 28232442 10.1128/genomeA.01648-16 PMC5323621

[mbt213270-bib-0044] Geerligs, G. , Aldrich, H.C. , Harder, W. , and Diekert, G. (1987) Isolation and characterization of a carbon monoxide utilizing strain of the acetogen *Peptostreptococcus productus* . Arch Microbiol 148: 305–313. https://doi.org/10.1007/BF00456709

[mbt213270-bib-0045] Genthner, B.S. , and Bryant, M.P. (1982) Growth of *Eubacterium limosum* with carbon monoxide as the energy source. Appl Environ Microbiol 43: 70–74. 16345931 10.1128/aem.43.1.70-74.1982 PMC241782

[mbt213270-bib-0046] Genthner, B.R. , Davis, C.L. , and Bryant, M.P. (1981) Features of rumen and sewage sludge strains of *Eubacterium limosum*, a methanol‐ and H_2_‐CO_2_‐utilizing species. Appl Environ Microbiol 42: 12–19. 6791591 10.1128/aem.42.1.12-19.1981 PMC243953

[mbt213270-bib-0047] Gerritsen, J. , Fuentes, S. , Grievink, W. , Niftrik, L.V. , Tindall, B.J. , Timmerman, H.M. , *et al* (2014) Characterization of *Romboutsia ilealis* gen. nov., sp. nov., isolated from the gastro‐intestinal tract of a rat, and proposal for the reclassification of five closely related members of the genus *Clostridium* into the genera *Romboutsia* gen. nov., *Intestinibacter* gen. nov., *Terrisporobacter* gen. nov. and *Asaccharospora* gen. nov. Int J Syst Evol Microbiol 64: 1600–1616. https://doi.org/10.1099/ijs.0.059543-0 24480908 10.1099/ijs.0.059543-0

[mbt213270-bib-0048] Gossner, A.S. (2006) Trophic interaction of the aerotolerant anaerobe *Clostridium intestinale* and the acetogen *Sporomusa rhizae* sp. nov. isolated from roots of the black needlerush *Juncus roemerianus* . Microbiol 152: 1209–1219. https://doi.org/10.1099/mic.0.28725-0 10.1099/mic.0.28725-0 16549683

[mbt213270-bib-0601] Gößner, A.S. , Devereux, R. , Ohnemüller, N. , Acker, G. , Stackebrandt, E. , and Drake, H.L. (1999) *Thermicanus aegyptius* gen. nov., sp. nov., isolated from oxic soil, a fermentative microaerophile that grows commensally with the thermophilic acetogen *Moorella thermoacetica* . Appl Environ Microbiol 65: 5124–5133. 10543831 10.1128/aem.65.11.5124-5133.1999 PMC91689

[mbt213270-bib-0602] Gößner, A.S. , Küsel, K. , Schulz, D. , Trenz, S. , Acker, G. , Lovell, C.R. , *et al* (2006) Trophic interaction of the aerotolerant anaerobe *Clostridium intestinale* and the acetogen *Sporomusa rhizae* sp. nov. isolated from roots of the black needlerush *Juncus roemerianus* . Microbiology 152: 1209–1219. 16549683 10.1099/mic.0.28725-0

[mbt213270-bib-0049] Gößner, A.S. , Picardal, F. , Tanner, R.S. , and Drake, H.L. (2008) Carbon metabolism of the moderately acid‐tolerant acetogen *Clostridium drakei* isolated from peat. FEMS Microbiol Lett 287: 236–242. https://doi.org/10.1111/j.1574-6968.2008.01313.x 18710398 10.1111/j.1574-6968.2008.01313.x

[mbt213270-bib-0050] Gottwald, M. , Andreesen, J.R. , LeGall, J. , and Ljungdahl, L.G. (1975) Presence of cytochrome and menaquinone in *Clostridium formicoaceticum* and *Clostridium thermoaceticum* . J Bacteriol 122: 325–328. 1123319 10.1128/jb.122.1.325-328.1975 PMC235673

[mbt213270-bib-0603] Graber, J.R. , and Breznak, J.A. (2004) Physiology and nutrition of *Treponema primitia*, an H_2_/CO_2_‐acetogenic spirochete from termite hindguts. Appl Environ Microbiol 70: 1307–1314. 15006747 10.1128/AEM.70.3.1307-1314.2004 PMC368360

[mbt213270-bib-0604] Graber, J.R. , Leadbetter, J.R. , and Breznak, J.A. (2004) Description of *Treponema azotonutricium* sp. nov. and *Treponema primitia* sp. nov., the first spirochetes isolated from termite guts. Appl Environ Microbiol 70: 1315–1320. 15006748 10.1128/AEM.70.3.1315-1320.2004 PMC368361

[mbt213270-bib-0051] Greening, R.C. , and Leedle, J.A. (1989) Enrichment and isolation of *Acetitomaculum ruminis*, gen. nov., sp. nov.: acetogenic bacteria from the bovine rumen. Arch Microbiol 151: 399–406. https://doi.org/10.1007/BF00416597 2500921 10.1007/BF00416597

[mbt213270-bib-0052] Griffin, D.W. , and Schultz, M.A. (2012) Fuel and chemical products from biomass syngas: a comparison of gas fermentation to thermochemical conversion routes. Environ Prog & Sustain Ener 31: 219–224.

[mbt213270-bib-0053] Groher, A. , and Weuster‐Botz, D. (2016a) Comparative reaction engineering analysis of different acetogenic bacteria for gas fermentation. J Biotechnol 228: 82–94. 27107467 10.1016/j.jbiotec.2016.04.032

[mbt213270-bib-0054] Groher, A. , and Weuster‐Botz, D. (2016b) General medium for the autotrophic cultivation of acetogens. Bioproc Biosys Eng 39: 1645–1650. 10.1007/s00449-016-1634-5 27270418

[mbt213270-bib-0055] Heijstra, B.D. , Leang, C. , and Juminaga, A. (2017) Gas fermentation: cellular engineering possibilities and scale‐up. Microb Cell Fact 16: 60. 28403896 10.1186/s12934-017-0676-y PMC5389167

[mbt213270-bib-0056] Heinrich, D. , Raberg, M. and Steinbüchel, A. (2017) Studies on the aerobic utilization of synthesis gas (syngas) by wild type and recombinant strains of *Ralstonia eutropha* H16. Microbial Biotechnol. https://doi.org/10.1111/1751-7915.12873 10.1111/1751-7915.12873 PMC6011924 29027357

[mbt213270-bib-0057] Hermann, M. , Popoff, M. , and Sebald, M. (1987) *Sporomusa paucivorans* sp. nov., a methylotrophic bacterium that forms acetic acid from hydrogen and carbon dioxide. Int J Syst Bacteriol 37: 93–101. https://doi.org/10.1099/00207713-37-2-93

[mbt213270-bib-0058] Hess, V. , Schuchmann, K. , and Müller, V. (2013) The ferredoxin: NAD^+^ oxidoreductase (Rnf) from the acetogen *Acetobacterium woodii* requires Na^+^ and is reversibly coupled to the membrane potential. J Biol Chem 288: 31496–31502. https://doi.org/10.1074/jbc.M113.510255 24045950 10.1074/jbc.M113.510255 PMC3814746

[mbt213270-bib-0059] Hoffmeister, S. , Gerdom, M. , Bengelsdorf, F.R. , Linder, S. , Flüchter, S. , Öztürk, H. , *et al* (2016) Acetone production with metabolically engineered strains of *Acetobacterium woodii* . Metabol Eng 36: 37–47. https://doi.org/10.1016/j.ymben.2016.03.001 10.1016/j.ymben.2016.03.001 26971669

[mbt213270-bib-0060] Huhnke, R.L. , Lewis, R.S. and Tanner, R.S. (2008) Isolation and characterization of novel clostridial species. US20080057554 A1. Washington, DC: U.S. Patent and Trademark Office.

[mbt213270-bib-0061] Humphreys, C.M. , Mclean, S. , Schatschneider, S. , Millat, T. , Henstra, A.M. , Annan, F.J. , *et al* (2015) Whole genome sequence and manual annotation of *Clostridium autoethanogenum*, an industrially relevant bacterium. BMC Genom 16: 1085 https://doi.org/10.1186/s12864-015-2287-5 10.1186/s12864-015-2287-5 PMC4687164 26692227

[mbt213270-bib-0062] Humphreys, J.R. , Daniel, R. , and Poehlein, A. (2017a) Genome sequence of the homoacetogenic, gram‐negative, endospore‐forming bacterium *Sporomusa acidovorans* Mol DSM 3132. Genome Announc 5: e00983–17. https://doi.org/10.1128/genomea.00983-17 28935740 10.1128/genomeA.00981-17 PMC5609419

[mbt213270-bib-0063] Humphreys, J.R. , Daniel, R. , and Poehlein, A. (2017b) Insights into the genome of the anaerobic acetogen *Sporomusa silvacetica* DG‐1 DSM 10669. Genome Announc 5: e00981–17. https://doi.org/10.1128/genomea.00981-17 28935741 10.1128/genomeA.00983-17 PMC5609420

[mbt213270-bib-0064] Hwang, S. , Song, Y. , and Cho, B. (2015) Draft genome sequence of *Acetobacterium bakii* DSM 8239, a potential psychrophilic chemical producer through syngas fermentation. Genome Announc 3: e01070–15. https://doi.org/10.1128/genomea.01070-15 26404601 10.1128/genomeA.01070-15 PMC4582577

[mbt213270-bib-0065] Jeong, Y. , Song, Y. , Shin, H.S. , and Cho, B. (2014) Draft genome sequence of acid‐tolerant *Clostridium drakei* SL1^T^, a potential chemical producer through syngas fermentation. Genome Announc 2: e00387–14. https://doi.org/10.1128/genomea.00387-14 24831144 10.1128/genomeA.00387-14 PMC4022808

[mbt213270-bib-0066] Kane, M.D. , and Breznak, J.A. (1991) *Acetonema longum* gen. nov. sp. nov., an H_2_/CO_2_ acetogenic bacterium from the termite. Pterotermes occidentis. Arch Microbiol 156: 91–98. https://doi.org/10.1007/BF00290979 1723588 10.1007/BF00290979

[mbt213270-bib-0067] Kane, M.D. , Brauman, A. , and Breznak, J.A. (1991) *Clostridium mayombei* sp. nov., an H_2_/CO_2_ acetogenic bacterium from the gut of the african soil‐feeding termite, *Cubitermes speciosus* . Arch Microbiol 156: 99–104.

[mbt213270-bib-0068] Kaneuchi, C. , Benno, Y. , and Mitsuoka, T. (1976) *Clostridium coccoides*, a new species from the feces of mice. Int J Syst Bacteriol 26: 482–486. https://doi.org/10.1099/00207713-26-4-482

[mbt213270-bib-0069] Kantzow, C. , and Weuster‐Botz, D. (2016) Effects of hydrogen partial pressure on autotrophic growth and product formation of *Acetobacterium woodii* . Bioproc Biosys Eng 39: 1325–1330. 10.1007/s00449-016-1600-2 27059835

[mbt213270-bib-0070] Kantzow, C. , Mayer, A. , and Weuster‐Botz, D. (2015) Continuous gas fermentation by *Acetobacterium woodii* in a submerged membrane reactor with full cell retention. J Biotechnol 212: 11–18. 26239230 10.1016/j.jbiotec.2015.07.020

[mbt213270-bib-0071] Karl, M.M. , Poehlein, A. , Bengelsdorf, F.R. , Daniel, R. , and Dürre, P. (2017) Complete genome sequence of the autotrophic acetogen *Clostridium formicaceticum* DSM 92^T^ using Nanopore and Illumina sequencing data. Genome Announc 5: e00423–17. https://doi.org/10.1128/genomea.00423-17 28546497 10.1128/genomeA.00423-17 PMC5477410

[mbt213270-bib-0607] Kerby, R. , and Zeikus, J.G. (1983) Growth of *Clostridium thermoaceticum* on H_2_/CO_2_ or CO as energy source. Curr Microbiol 8: 27–30.

[mbt213270-bib-0072] King, G.M. (2003) Molecular and culture‐based analyses of aerobic carbon monoxide oxidizer diversity. Appl Environ Microbiol 69: 7257–7265. 14660374 10.1128/AEM.69.12.7257-7265.2003 PMC309980

[mbt213270-bib-0073] King, G.M. , and Weber, C.F. (2007) Distribution, diversity and ecology of aerobic CO‐oxidizing bacteria. Nat Rev Microbiol 5: 107–118. 17224920 10.1038/nrmicro1595

[mbt213270-bib-0074] Köpke, M. , Held, C. , Hujer, S. , Liesegang, H. , Wiezer, A. , Wollherr, A. , *et al* (2010) *Clostridium ljungdahlii* represents a microbial production platform based on syngas. Proc Natl Acad Sci USA 107: 13087–13092. https://doi.org/10.1073/pnas.1004716107 20616070 10.1073/pnas.1004716107 PMC2919952

[mbt213270-bib-0075] Köpke, M. , Mihalcea, C. , Liew, F. , Tizard, J.H. , Ali, M.S. , Conolly, J.J. , *et al* (2011) 2,3‐butanediol production by acetogenic bacteria, an alternative route to chemical synthesis, using industrial waste gas. Appl Environ Microbiol 77: 5467–5475. https://doi.org/10.1128/AEM.00355-11 21685168 10.1128/AEM.00355-11 PMC3147483

[mbt213270-bib-0076] Köpke, M. , Straub, M. , and Dürre, P. (2013) *Clostridium difficile* is an autotrophic bacterial pathogen. PLoS ONE 8: 4 https://doi.org/10.1371/journal.pone.0062157 10.1371/journal.pone.0062157 PMC3633928 23626782

[mbt213270-bib-0077] Kotsyurbenko, O.R. , Simankova, M.V. , Available, A.N. , Zhilina, T.N. , Bolotina, N.P. , Lysenko, A.M. , and Osipov, G.A. (1995) New species of psychrophilic acetogens: *Acetobacterium bakii* sp. nov., *A. paludosum* sp. nov. A. fimetarium sp. nov. Arch Microbiol 163: 29–34. https://doi.org/10.1007/s002030050167

[mbt213270-bib-0078] Krumholz, L.R. , and Bryant, M.P. (1985) *Clostridium pfennigii* sp. nov. uses methoxyl groups of monobenzenoids and produces butyrate. Int J Syst Bacteriol 35: 454–456. https://doi.org/10.1099/00207713-35-4-454

[mbt213270-bib-0079] Kuhner, C.H. , Frank, C. , Griesszhammer, A. , Schmittroth, M. , Acker, G. , Gosszner, A. , and Drake, H.L. (1997) *Sporomusa silvacetica* sp. nov., an acetogenic bacterium isolated from aggregated forest soil. Int J Syst Bacteriol 47: 352–358. https://doi.org/10.1099/00207713-47-2-352 9103621 10.1099/00207713-47-2-352

[mbt213270-bib-0080] Küsel, K. , Dorsch, T. , Acker, G. , Stackebrandt, E. , and Drake, H.L. (2000) *Clostridium scatologenes* strain SL1 isolated as an acetogenic bacterium from acidic sediments. Int J Syst Evol Microbiol 50: 537–546. https://doi.org/10.1099/00207713-50-2-537 10758858 10.1099/00207713-50-2-537

[mbt213270-bib-0081] Küsel, K. , Karnholz, A. , Trinkwalter, T. , Devereux, R. , Acker, G. , and Drake, H.L. (2001) Physiological ecology of *Clostridium glycolicum* RD‐1, an aerotolerant acetogen isolated from sea grass roots. Appl Environ Microbiol 67: 4734–4741. https://doi.org/10.1128/AEM.67.10.4734-4741.2001 11571179 10.1128/AEM.67.10.4734-4741.2001 PMC93226

[mbt213270-bib-0082] LanzaTech (2017) From trash to tank: upcycling from landfill to fuel demonstrated in Japan. URL http://www.lanzatech.com/trash-tank-upcycling-landfill-fuel-demonstrated-japan/.

[mbt213270-bib-0083] Latif, H. , Zeidan, A.A. , Nielsen, A.T. , and Zengler, K. (2014) Trash to treasure: production of biofuels and commodity chemicals via syngas fermenting microorganism. Curr Opin Biotechnol 27: 79–87. 24863900 10.1016/j.copbio.2013.12.001

[mbt213270-bib-0084] Le Quéré, C. , Andrew, R.M. , Friedlingstein, P. , Sitch, S. , Pongratz, J. , Manning, A.C. , *et al* (2017) Global carbon budget 2017. Earth Syst Sci Data Discuss 8, 859–861. https://doi.org/10.5194/essdd-2017-123

[mbt213270-bib-0085] Liesack, W. , Bak, F. , Kreft, J. , and Stackebrandt, E. (1994) *Holophaga foetida* gen. nov., sp. nov., a new, homoacetogenic bacterium degrading methoxylated aromatic compounds. Arch Microbiol 162: 85–90. https://doi.org/10.1007/bf00264378 8085918 10.1007/BF00264378

[mbt213270-bib-0086] Liguori, R. , and Faraco, V. (2016) Biological processes for advancing lignocellulosic waste biorefinery by advocating circular economy. Bioresour Technol 215: 13–20. 27131870 10.1016/j.biortech.2016.04.054

[mbt213270-bib-0087] Liou, J.S.C. , Balkwill, D.L. , Drake, G.R. , and Tanner, R.S. (2005) *Clostridium carboxidivorans* sp. nov., a solvent‐producing clostridium isolated from an agricultural settling lagoon, and reclassification of the acetogen *Clostridium scatologenes* strain SL1 as *Clostridium drakei* sp. nov. Int J Syst Evol Microbiol 55: 2085–2091. https://doi.org/10.1099/ijs.0.63482-0 16166714 10.1099/ijs.0.63482-0

[mbt213270-bib-0088] Liu, C. , Finegold, S.M. , Song, Y. , and Lawson, P.A. (2008) Reclassification of *Clostridium coccoides*,* Ruminococcus hansenii*,* Ruminococcus hydrogenotrophicus*,* Ruminococcus luti*,* Ruminococcus productus* and *Ruminococcus schinkii* as *Blautia coccoides* gen. nov., comb. nov., *Blautia hansenii* comb. nov., *Blautia hydrogenotrophica* comb. nov., *Blautia luti* comb. nov., *Blautia product*a comb. nov., *Blautia schinkii* comb. nov. and description of *Blautia wexlerae* sp. nov., isolated from human faeces. Int J Syst Evol Microbiol 58: 1896–1902. https://doi.org/10.1099/ijs.0.65208-0 18676476 10.1099/ijs.0.65208-0

[mbt213270-bib-0089] Liu, C. , Li, J. , Zhang, Y. , Philip, A. , Shi, E. , Chi, X. , and Meng, J. (2015) Influence of glucose fermentation on CO_2_ assimilation to acetate in homoacetogen *Blautia coccoides* GA‐1. J Ind Microbiol Biotechnol 42: 1217–1224. https://doi.org/10.1007/s10295-015-1646-1 26153502 10.1007/s10295-015-1646-1

[mbt213270-bib-0090] Lorowitz, W.H. , and Bryant, M.P. (1984) *Peptostreptococcus productus* strain that grows rapidly with CO as the energy source. Appl Environ Microbiol 47: 961–964. 6430231 10.1128/aem.47.5.961-964.1984 PMC240027

[mbt213270-bib-0091] Lux, M.F. , and Drake, H.L. (1992) Re‐examination of thee metabolic potentials of the acetogens *Clostridium aceticum* and *Clostridium formicoaceticum*: chemolithoautotrophic and aromatic‐dependent growth. FEMS Microbiol Lett 95: 49–56. https://doi.org/10.1111/j.1574-6968.1992.tb05341.x 10.1016/0378-1097(92)90735-7 1516807

[mbt213270-bib-0092] Lynd, L. , Kerby, R. , and Zeikus, J.G. (1982) Carbon monoxide metabolism of the methylotrophic acidogen *Butyribacterium methylotrophicum* . J Bacteriol 149: 255–263. 7033210 10.1128/jb.149.1.255-263.1982 PMC216617

[mbt213270-bib-0093] Martin, M.E. , Richter, H. , Saha, S. , and Angenent, L.T. (2015) Traits of selected Clostridium strains for syngas fermentation to ethanol. Biotechnol Bioeng 113: 531–539. 26331212 10.1002/bit.25827

[mbt213270-bib-0094] Mayer, A. and Weuster‐Botz, D. (2017) Reaction engineering analysis of the autotrophic energy metabolism of *Clostridium aceticum* . FEMS Microbiol Lett 364, fnx219 https://doi.org/10.1093/femsle/fnx219 10.1093/femsle/fnx219 29069379

[mbt213270-bib-0095] Mechichi, T. , Labat, M. , Woo, T.H. , Thomas, P. , Garcia, J. , and Patel, B.K. (1998) *Eubacterium aggregans* sp. nov., a new homoacetogenic bacterium from olive mill wastewater treatment digester. Anaerobe 4: 283–291. https://doi.org/10.1006/anae.1998.0179 16887654 10.1006/anae.1998.0179

[mbt213270-bib-0096] Mechichi, T. , Labat, M. , Patel, B.K. , Woo, T.H. , Thomas, P. , and Garcia, J. (1999) *Clostridium methoxybenzovorans* sp. nov., a new aromatic o‐demethylating homoacetogen from an olive mill wastewater treatment digester. Int J Syst Bacteriol 49: 1201–1209. https://doi.org/10.1099/00207713-49-3-1201 10425780 10.1099/00207713-49-3-1201

[mbt213270-bib-0097] Meyer, O. , and Schlegel, H.G. (1978) Reisolation of the carbon monoxide utilizing hydrogen bacterium *Pseudomonas carboxydovorans* (Kistner) comb. nov. Arch Microbiol 118: 35–43. 697501 10.1007/BF00406071

[mbt213270-bib-0098] Meyer, O. , and Schlegel, H.G. (1983) Biology of aerobic carbon monoxide‐oxidizing bacteria. Annu Rev Microbiol 37: 277–310. 6416144 10.1146/annurev.mi.37.100183.001425

[mbt213270-bib-0099] Mikkelsen, M. , Jørgensen, M. and Krebs, C.F. (2010) The teraton challenge. A review of fixation and transformation of carbon dioxide. Energy Environ Sci 3, 43–81.

[mbt213270-bib-0100] Mohammadi, M. , Younesi, H. , Najafpour, G. , and Mohamed, A.R. (2012) Sustainable ethanol fermentation from synthesis gas by *Clostridium ljungdahlii* in a continuous stirred tank bioreactor. J Chem Technol Biotechnol 87: 837–843.

[mbt213270-bib-0101] Mohammadi, M. , Mohamed, A.R. , Najafpour, G.D. , Younesi, H. , and Uzir, M.H. (2014) Kinetic studies on fermentative production of biofuel from synthesis gas using *Clostridium ljungdahlii* . Sci World J 2014: 910590. 10.1155/2014/910590 PMC3925604 24672390

[mbt213270-bib-0102] Molitor, B. , Richter, H. , Martin, M.E. , Jensen, R.O. , Juminaga, A. , Mihalcea, C. , and Angenent, L.T. (2016) Carbon recovery by fermentation of CO‐rich off gases – Turning steel mills into biorefineries. Biores Technol 215: 386–396. 10.1016/j.biortech.2016.03.094 27095410

[mbt213270-bib-0103] Möller, B. , Ossmer, R. , Howard, B.H. , Gottschalk, G. , and Hippe, H. (1984) *Sporomusa*, a new genus of gram‐negative anaerobic bacteria including *Sporomusa sphaeroides* spec. nov. and *Sporomusa ovata* spec. nov. Arch Microbiol 139: 388–396. https://doi.org/10.1007/BF00408385

[mbt213270-bib-0104] Müller, V. , Aufurth, S. , and Rahlfs, S. (2001) The Na^+^ cycle in *Acetobacterium woodii*: identification and characterization of a Na^+^ translocating F_1_F_0_‐ATPase with a mixed oligomer of 8 and 16 kDa proteolipids. Biochim Biophys Acta 1505: 108–120. https://doi.org/10.1016/S0005-2728(00)00281-4 11248193 10.1016/s0005-2728(00)00281-4

[mbt213270-bib-0105] Munasinghe, P.C. , and Khanal, S.K. (2010) Biomass‐derived syngas fermentation into biofuels: opportunities and challenges. Biores Technol 101: 5013–5022. 10.1016/j.biortech.2009.12.098 20096574

[mbt213270-bib-0106] Ollivier, B. , Cordruwisch, R. , Lombardo, A. , and Garcia, J. (1985) Isolation and characterization of *Sporomusa acidovorans* sp. nov., a methylotrophic homoacetogenic bacterium. Arch Microbiol 142: 307–310. https://doi.org/10.1007/BF00693409

[mbt213270-bib-0107] Omae, K. , Yoneda, Y. , Fukuyama, Y. , Yoshida, T. , and Sako, Y. (2017) Genomic analysis of *Calderihabitans maritimus* KKC1, a thermophilic, hydrogenogenic, carboxydotrophic bacterium isolated from marine sediment. Appl Environ Microbiol 83: e00832–17. https://doi.org/10.1128/aem.00832-17 28526793 10.1128/AEM.00832-17 PMC5514679

[mbt213270-bib-0608] Parekh, S. , and Cheryan, M. (1991) Production of acetate by mutant strains of *Clostridium thermoaceticum* . Appl Microbiol Biotechnol 36: 384–387.

[mbt213270-bib-0108] Paul, D. , Bridges, S. , Burgess, S.C. , Dandass, Y. , and Lawrence, M.L. (2008) Genome sequence of the chemolithoautotrophic bacterium *Oligotropha carboxidovorans* OM5^T^ . J Bacteriol 190: 5531–5532. 18539730 10.1128/JB.00614-08 PMC2493269

[mbt213270-bib-0109] Pérez‐Fortes, M. , Schöneberger, J.C. , Boulamanti, A. , and Tzimas, E. (2016) Methanol synthesis using captured CO_2_ as raw material: Techno‐economic and environmental assessment. Appl Energy 161: 718–732.

[mbt213270-bib-0110] Pfitzer, C. , Dahmen, N. , Tröger, N. , Weirich, F. , Sauer, J. , Günther, A. , and Müller‐Hagedorn, M. (2016) Fast pyrolysis of wheat straw in the Bioliq pilot plant. Ener and Fuels 30: 8047–8054.

[mbt213270-bib-0111] Philip, J. (2018) The bioeconomy, the challenge of the century of the policy makers. N Biotechnol. 25: 11–19. https://doi.org/10.1016/j.nbt.2017.04.004 10.1016/j.nbt.2017.04.004 28487094

[mbt213270-bib-0112] Pierce, E. , Xie, G. , Barabote, R.D. , Saunders, E. , Han, C.S. , Detter, J.C. , *et al* (2008) The complete genome sequence of *Moorella thermoacetica* (f. *Clostridium thermoaceticum*). Environ Microbiol 10: 2550–2573. https://doi.org/10.1111/j.1462-2920.2008.01679.x 18631365 10.1111/j.1462-2920.2008.01679.x PMC2575129

[mbt213270-bib-0114] Plugge, C.M. , Balk, M. , and Stams, A.J. (2002) *Desulfotomaculum thermobenzoicum* subsp. *thermosyntrophicum* subsp. nov., a thermophilic, syntrophic, propionate‐oxidizing, spore‐forming bacterium. Int J Syst Evol Microbiol 52: 391–399. https://doi.org/10.1099/00207713-52-2-391 11931147 10.1099/00207713-52-2-391

[mbt213270-bib-0115] Poehlein, A. , Schmidt, S. , Kaster, A. , Goenrich, M. , Vollmers, J. , Thürmer, A. , *et al* (2012) An ancient pathway combining carbon dioxide fixation with the generation and utilization of a sodium ion gradient for ATP synthesis. PLoS ONE 7: e33439 https://doi.org/10.1371/journal.pone.0033439 22479398 10.1371/journal.pone.0033439 PMC3315566

[mbt213270-bib-0116] Poehlein, A. , Gottschalk, G. , and Daniel, R. (2013) First insights into the genome of the Gram‐negative, endospore‐forming organism *Sporomusa ovata* strain H1 DSM 2662. Genome Announc 1: e00734–13. https://doi.org/10.1128/genomea.00734-13 24029766 10.1128/genomeA.00734-13 PMC3772150

[mbt213270-bib-0117] Poehlein, A. , Bengelsdorf, F.R. , Esser, C. , Schiel‐Bengelsdorf, B. , Daniel, R. , and Dürre, P. (2015a) Complete genome sequence of the type strain of the acetogenic bacterium *Moorella thermoacetica* DSM 521^T^ . Genome Announc 3: e01159–15. https://doi.org/10.1128/genomea.01159-15 26450731 10.1128/genomeA.01159-15 PMC4599090

[mbt213270-bib-0118] Poehlein, A. , Bengelsdorf, F.R. , Schiel‐Bengelsdorf, B. , Gottschalk, G. , Daniel, R. , and Dürre, P. (2015b) Complete genome sequence of Rnf‐ and cytochrome‐containing autotrophic acetogen *Clostridium aceticum* DSM 1496. Genome Announc 3: e00786–15. https://doi.org/10.1128/genomea.00786-15 26184942 10.1128/genomeA.00786-15 PMC4505130

[mbt213270-bib-0119] Poehlein, A. , Cebulla, M. , Ilg, M.M. , Bengelsdorf, F.R. , Schiel‐Bengelsdorf, B. , Whited, G. , *et al* (2015c) The complete genome sequence of Clostridium aceticum: a missing link between Rnf‐ and cytochrome‐containing autotrophic acetogens. mBio 6, 5 https://doi.org/10.1128/mbio.01168-15 10.1128/mBio.01168-15 PMC4600107 26350967

[mbt213270-bib-0120] Poehlein, A. , Bengelsdorf, F.R. , Schiel‐Bengelsdorf, B. , Daniel, R. , and Dürre, P. (2016) Genome sequence of the acetogenic bacterium *Acetobacterium wieringae* DSM 1911^T^ . Genome Announc 4: e01430–16. https://doi.org/10.1128/genomea.01430-16 28007862 10.1128/genomeA.01430-16 PMC5180390

[mbt213270-bib-0121] Richter, H. , Martin, M.E. , and Angenent, L.T. (2013) A two‐stage continuous fermentation system for conversion of syngas into ethanol. Energies 6: 3987–4000.

[mbt213270-bib-0122] Riedel, T. , Bunk, B. , Thürmer, A. , Spröer, C. , Brzuszkiewicz, E. , Abt, B. , *et al* (2015) Genome resequencing of the virulent and multidrug‐resistant reference strain *Clostridium difficile* 630. Genome Announc 3: e00276–15. https://doi.org/10.1128/genomea.00276-15 25858846 10.1128/genomeA.00276-15 PMC4392158

[mbt213270-bib-0123] Rieu‐Lesme, F. , Morvan, B. , Collins, M. , Fonty, G. , and Willems, A. (1996) A new H_2_/CO_2_‐using acetogenic bacterium from the rumen: description of *Ruminococcus schinkii* sp. nov. FEMS Microbiol Lett 140: 281–286. https://doi.org/10.1111/j.1574-6968.1996.tb08350.x 8764491 10.1016/0378-1097(96)00195-4

[mbt213270-bib-0611] Rosenthal, A.Z. , Matson, E.G. , Eldar, A. , and Leadbetter, J.R. (2011) RNA‐seq reveals cooperative metabolic interactions between two termite‐gut spirochete species in co‐culture. ISME J 5: 1133–1142. 21326336 10.1038/ismej.2011.3 PMC3146290

[mbt213270-bib-0125] Schink, B. (1984) *Clostridium magnum* sp. nov., a non‐autotrophic homoacetogenic bacterium. Arch Microbiol 137: 250–255. https://doi.org/10.1007/BF00414553

[mbt213270-bib-0126] Schink, B. , and Stieb, M. (1983) Fermentative degradation of polyethylene glycol by a strictly anaerobic, Gram‐negative, non‐sporeforming bacterium, *Pelobacter venetianus* sp. nov. Appl Environ Microbiol 45: 1905–1913. 6881964 10.1128/aem.45.6.1905-1913.1983 PMC242557

[mbt213270-bib-0127] Schuchmann, K. , and Müller, V. (2014) Autotrophy at the thermodynamic limit of life: a model for energy conservation in acetogenic bacteria. Nat Rev Microbiol 12: 809–821. https://doi.org/10.1038/nrmicro3365 25383604 10.1038/nrmicro3365

[mbt213270-bib-0128] Schulte, M.J. , Wiltgen, J. , Ritter, J. , Mooney, C.B. , and Flickinger, M.C. (2016) A high gas fraction, reduced power, syngas bioprocessing method demonstrated with a *Clostridium ljungdahlii* OTA1 paper biocomposite. Biotechnol Bioeng 113: 1913–1923. 26927418 10.1002/bit.25966 PMC5810353

[mbt213270-bib-0129] Schuppert, B. , and Schink, B. (1990) Fermentation of methoxyacetate to glycolate and acetate by newly isolated strains of *Acetobacterium* sp. Arch Microbiol 153: 200–204. https://doi.org/10.1007/BF00247821

[mbt213270-bib-0130] Shen, Y. , Brauwn, R.C. , and Wen, Z. (2017) Syngas fermentation by *Clostridium carboxidivorans* P7 in a horizontal rotating packed bed biofilm reactor with enhanced ethanol production. Appl Energy 187: 585–594.

[mbt213270-bib-0131] Sikorski, J. , Lapidus, A. , Chertkov, O. , Lucas, S. , Copeland, A. , Rio, T.G. , *et al* (2010) Complete genome sequence of *Acetohalobium arabaticum* type strain (Z‐7288^T^). Stand Genomic Sci 3, 57–65. https://doi.org/10.4056/sigs.1062906 21304692 10.4056/sigs.1062906 PMC3035264

[mbt213270-bib-0132] Simankova, M.V. , Kotsyurbenko, O.R. , Stackebrandt, E. , Kostrikina, N.A. , Lysenko, A.M. , Osipov, G.A. , and Nozhevnikova, A.N. (2000) *Acetobacterium tundrae* sp. nov., a new psychrophilic acetogenic bacterium from tundra soil. Arch Microbiol 174: 440–447. https://doi.org/10.1007/s002030000229 11195100 10.1007/s002030000229

[mbt213270-bib-0133] Skidmore, B.E. , Baker, R.A. , Banjade, D.R. , Bray, J.M. , Tree, D.R. , and Lewis, R.S. (2013) Syngas fermentation to biofuels: effects of hydrogen partial pressure on hydrogenase efficiency. Biomass Bioenerg 55: 165–162.

[mbt213270-bib-0134] Sleat, R. , Mah, R.A. , and Robinson, R. (1985) *Acetoanaerobium noterae* gen. nov., sp. nov.: an anaerobic bacterium that forms acetate from H_2_ and CO_2_ . Int J Syst Bacteriol 35: 10–15. https://doi.org/10.1099/00207713-35-1-10

[mbt213270-bib-0609] Slobodkin, A. , Reysenbach, A. , Mayer, F. , and Wiegel, J. (1997) Isolation and characterization of the homoacetogenic thermophilic bacterium *Moorella glycerini* sp. nov. Int J Syst Bacteriol 47: 969–974. 9336894 10.1099/00207713-47-4-969

[mbt213270-bib-0135] Song, Y. , and Cho, B. (2015) Draft genome sequence of chemolithoautotrophic acetogenic butanol‐producing *Eubacterium limosum* ATCC 8486. Genome Announc 3: e01564–14. https://doi.org/10.1128/genomea.01564-14 25676768 10.1128/genomeA.01564-14 PMC4333668

[mbt213270-bib-0136] Strätz, M. , Sauer, U. , Kuhn, A. , and Dürre, P. (1994) Plasmid transfer into the homoacetogen *Acetobacterium woodii* by electroporation and conjugation. Appl Environ Microbiol 60: 1033–1037. 16349209 10.1128/aem.60.3.1033-1037.1994 PMC201430

[mbt213270-bib-0137] Straub, M. , Demler, M. , Weuster‐Botz, D. , and Dürre, P. (2014) Selective enhancement of autotrophic acetate production with genetically modified *Acetobacterium woodii* . J Biotechnol 178: 67–72. https://doi.org/10.1016/j.jbiotec.2014.03.005 24637370 10.1016/j.jbiotec.2014.03.005

[mbt213270-bib-0610] Svetlichny, V. , Sokolova, T. , Gerhardt, M. , Ringpfeil, M. , Kostrikina, N. , and Zavarzin, G. (1991) *Carboxydothermus hydrogenoformans* gen. nov., sp. nov., a CO‐utilizing thermophilic anaerobic bacterium from hydrothermal environments of Kunashir Island. Syst Appl Microbiol 14: 254–260.

[mbt213270-bib-0138] Takors, R. (2012) Scale‐up of microbial processes: Impacts, tools and open questions. J Biotechnol 160: 3–9. 22206982 10.1016/j.jbiotec.2011.12.010

[mbt213270-bib-0139] Tanaka, K. , and Pfennig, N. (1988) Fermentation of 2‐methoxyethanol by *Acetobacterium malicum* sp. nov. and *Pelobacter venetianus* . Arch Microbiol 149: 181–187. https://doi.org/10.1007/BF00422003

[mbt213270-bib-0140] Tanner, R.S. , Miller, L.M. , and Yang, D. (1993) *Clostridium ljungdahlii* sp. nov., an acetogenic species in clostridial rRNA homology group I. Int J Syst Bacteriol 43: 232–236. https://doi.org/10.1099/00207713-43-2-232 7684239 10.1099/00207713-43-2-232

[mbt213270-bib-0141] Traunecker, J. , Preuß, A. , and Diekert, G. (1991) Isolation and characterization of a methyl chloride utilizing, strictly anaerobic bacterium. Arch Microbiol 156: 416–421. https://doi.org/10.1007/BF00248720

[mbt213270-bib-0142] Tremblay, P. , Zhang, T. , Dar, S.A. , Leang, C. and Lovley, D.R. (2012) The Rnf complex of *Clostridium ljungdahlii* is a proton‐translocating ferredoxin: NAD+ oxidoreductase essential for autotrophic growth. mBio 4, 1 https://doi.org/10.1128/mbio.00406-12 10.1128/mBio.00406-12 PMC3531802 23269825

[mbt213270-bib-0144] Uhlig, R. , Poehlein, A. , Fischer, R. , Daniel, R. , and Bahl, H. (2016) Genome sequence of the autotrophic acetogen *Clostridium magnum* DSM 2767. Genome Announc 4: e00464–16. https://doi.org/10.1128/genomea.00464-16 27284147 10.1128/genomeA.00464-16 PMC4901216

[mbt213270-bib-0145] Vega, J.L. , Holmberg, V.L. , Clausen, E.C. , and Gaddy, J.L. (1988) Fermentation parameters of *Peptostreptococcus productus* on gaseous substrates (CO, H_2_/CO_2_). Arch Microbiol 151: 65–70.

[mbt213270-bib-0148] Volland, S. , Rachinger, M. , Strittmatter, A. , Daniel, R. , Gottschalk, G. , and Meyer, O. (2011) Complete genome sequences of the chemolithoautotrophic *Oligotropha carboxidovorans* strains OM4 and OM5. J Bacteriol 193: 5043. 21742883 10.1128/JB.05619-11 PMC3165685

[mbt213270-bib-0149] Weber, C.F. , and King, G.M. (2012) The phylogenetic distribution and ecological role of carbon monoxide oxidation in the genus *Burkholderia* . FEMS Microbiol Ecol 79: 167–175. 22029898 10.1111/j.1574-6941.2011.01206.x

[mbt213270-bib-0150] Wiegel, J. , Braun, M. , and Gottschalk, G. (1981) *Clostridium thermoautotrophicum* species novum, a thermophile producing acetate from molecular hydrogen and carbon dioxide. Curr Microbiol 5: 255–260. https://doi.org/10.1007/BF01571158

[mbt213270-bib-0151] Wieringa, K.T. (1936) Over het verdwijnen van waterstof en koolzuur onder anaerobe voorwaarden. Ant Leeuwenhoek 3: 263–273. https://doi.org/10.1007/BF02059556

[mbt213270-bib-0152] Wolin, M.J. , Miller, T.L. , Collins, M.D. , and Lawson, P.A. (2003) Formate‐dependent growth and homoacetogenic fermentation by a bacterium from human feces: description of *Bryantella formatexigens* gen. nov., sp. nov. Appl Environ Microbiol 69: 6321–6326. https://doi.org/10.1128/AEM.69.10.6321-6326.2003 14532100 10.1128/AEM.69.10.6321-6326.2003 PMC201199

[mbt213270-bib-0153] Wolin, M.J. , Miller, T.L. and Lawson, P.A. (2008) Proposal to replace the illegitimate genus name *Bryantella* Wolin *et al*. 2004^VP^ with the genus name *Marvinbryantia* gen. nov. and to replace the illegitimate combination *Bryantella formatexigens* Wolin *et al*. 2004^VP^ with *Marvinbryantia formatexigens* comb. nov. Int J Syst Evol Microbiol 58, 742–744. https://doi.org/10.1099/ijs.0.65850-0 18319487 10.1099/ijs.0.65850-0

[mbt213270-bib-0612] Wu, M. , Ren, Q. , Durkin, A.S. , Daugherty, S.C. , Brinkac, L.M. , Dodson, R.J. , *et al* (2005) Life in hot carbon monoxide: The complete genome sequence of *Carboxydothermus hydrogenoformans* Z‐2901. PLoS Genet 1: 5. 10.1371/journal.pgen.0010065 PMC1287953 16311624

[mbt213270-bib-0154] Yasin, M. , Jeong, Y. , Park, S.J. , Jeong, J. , Lee, E.Y. , Lowitt, R.W. , *et al* (2015) Microbial synthesis gas utilization and ways to resolve kinetic and mass‐transfer limitations. Biores Technol 177: 361–374. 10.1016/j.biortech.2014.11.022 25443672

[mbt213270-bib-0615] Yoneda, Y. , Yoshida, T. , Kawaichi, S. , Daifuku, T. , Takabe, K. , and Sako, Y. (2012) *Carboxydothermus pertinax* sp. nov., a thermophilic, hydrogenogenic, Fe(III)‐reducing, sulfur‐reducing carboxydotrophic bacterium from an acidic hot spring. Int J Syst Evol Microbiol 62: 1692–1697. 21908679 10.1099/ijs.0.031583-0

[mbt213270-bib-0155] Zahn, J.A. and Saxena, J. (2012) Ethanologenic Clostridium species, Clostridium coskatii. Patent US 8143037 B2. Washington, DC: U.S. Patent and Trademark Office.

[mbt213270-bib-0156] Zavarzin, G.A. , and Nozhevnikova, A.N. (1977) Aerobic carboxydobacteria. Microb Ecol 3: 305–326. 24233667 10.1007/BF02010738

[mbt213270-bib-0157] Zeikus, J.G. , Lynd, L.H. , Thompson, T.E. , Krzycki, J.A. , Weimer, P.J. , and Hegge, P.W. (1980) Isolation and characterization of a new, methylotrophic, acidogenic anaerobe, the marburg strain. Curr Microbiol 3: 381–386. https://doi.org/10.1007/BF02601907

[mbt213270-bib-0158] Zhilina, T.N. , and Zavarzin, G.A. (1990) Extremely halophilic, methylotrophic, anaerobic bacteria. FEMS Microbiol Lett 87: 315–321. https://doi.org/10.1016/0378-1097(90)90472-3

[mbt213270-bib-0159] Zhilina, T.N. , Zavarzina, D.G. , Panteleeva, A.N. , Osipov, G.A. , Kostrikina, N.A. , Tourova, T.P. , and Zavarzin, G.A. (2012) *Fuchsiella alkaliacetigena* gen. nov., sp. nov., an alkaliphilic, lithoautotrophic homoacetogen from a soda lake. Int J Syst Evol Microbiol 62: 1666–1673. https://doi.org/10.1099/ijs.0.034363-0 21908678 10.1099/ijs.0.034363-0

[mbt213270-bib-0160] Zhilina, T.N. , Kuznetsov, B.B. , Zavarzina, D.G. , Detkova, E.N. , and Patutina, E.O. (2015) *Fuchsiella ferrireducens* sp. nov., a novel haloalkaliphilic, lithoautotrophic homoacetogen capable of iron reduction, and emendation of the description of the genus *Fuchsiella* . Int J Syst Evol Microbiol 65: 2432–2440. https://doi.org/10.1099/ijs.0.000278 25908709 10.1099/ijs.0.000278

[mbt213270-bib-0161] Zhu, Z. , Guo, T. , Zheng, H. , Song, T. , Ouyang, P. , and Xie, J. (2015) Complete genome sequence of a malodorant‐producing acetogen, *Clostridium scatologenes* ATCC 25775^T^ . J Biotechnol 212: 19–20. https://doi.org/10.1016/j.jbiotec.2015.07.013 26210291 10.1016/j.jbiotec.2015.07.013

